# Cilengitide limits syndromic and sporadic thoracic aortic aneurysm by targeting specific αV integrin

**DOI:** 10.1084/jem.20260700

**Published:** 2026-11-11

**Authors:** Sara Rega, Silvia Bouhuis, Maddalena Brusati, Tea Maverna, Nadia Fanotti, Manuel Casaburo, Simone Vodret, Francesco Riccitelli, Federico Celotto, Luca Lambroia, Montserrat Climent, Leonardo Elia, Martina Vescio, Linda Pattini, Rosaria Santoro, Veronika Myasoedova, Paolo Poggio, Giorgia Bonalumi, Nathasha Samali Udugampolage, Jacopo Taurino, Alessandro Pini, Serena Zacchigna, Giulio Pompilio, Gianluca L. Perrucci

**Affiliations:** 1 Unit of Cardio-Oncology and Vascular Biology, Centro Cardiologico Monzino IRCCS, Milan, Italy; 2 Animal Facility, Centro Cardiologico Monzino IRCCS, Milan, Italy; 3 Cardiovascular Biology Laboratory, International Centre for Genetic Engineering and Biotechnology, Trieste, Italy; 4 Bioinformatics Unit, IRCCS Humanitas Research Hospital, Milan, Italy; 5 Department of Biomedical Sciences, Humanitas University, Milan, Italy; 6 Humanitas Cardio Center, IRCCS Humanitas Research Hospital, Milan, Italy; 7 Department of Molecular and Translational Medicine, University of Brescia, Brescia, Italy; 8 Cardio-Tech Lab, Centro Cardiologico Monzino IRCCS, Milan, Italy; 9 Department of Electronics, Information and Bioengineering, Politecnico di Milano, Milan, Italy; 10 Unit of Inherited Cardiomyopathy, Centro Cardiologico Monzino IRCCS, Milan, Italy; 11 Unit of Applied Biotechnology in Cardiovascular Inflammation, Centro Cardiologico Monzino IRCCS, Milan, Italy; 12 Department of Biomedical, Surgical and Dental Sciences, Università degli Studi di Milano, Milan, Italy; 13 Department of Cardiovascular Surgery, Centro Cardiologico Monzino IRCCS, Milan, Italy; 14 Centro di Cardio Genetica Vascolare, IRCCS Policlinico San Donato, Milan, Italy; 15 Department of Medicine, Surgery and Health Sciences, Università degli Studi di Trieste, Trieste, Italy

## Abstract

Thoracic aortic aneurysm (TAA) is one of the major causes of morbidity and mortality worldwide, still lacking an etiologic therapy. Aberrant activation of TGF-β and dysregulation of mechanotransduction signalling in vascular smooth muscle cells (VSMCs) play a key role in the pathology. However, a precise picture of molecular mechanisms in the disease is still unclear. Integrins containing the αV subunit are central in pro-fibrotic pathways mediated by TGF-β and mechanotransduction signalling pathways, but their role as a potential therapeutic target in syndromic and sporadic TAAs still needs investigation. We showed higher expression levels of αV integrin in both types of human TAAs and that the αV integrin inhibitor Cilengitide limits pro-fibrotic events in patient-derived VSMCs. In vivo results defined Cilengitide as the most effective and promising drug for the treatment of TAAs in humans.

## Teaser

Rega et al. show that selective inhibition of αVβ3/αVβ5 integrins with Cilengitide reduces profibrotic signalling and aortic dilation in syndromic and sporadic thoracic aortic aneurysm models, identifying αV integrins as therapeutic targets and supporting Cilengitide repurposing for aortic disease.

## Introduction

Thoracic aortic aneurysm (TAA) consists of a weakened and dilated portion of the thoracic aorta, often resulting in more than a 50% increase in the aortic diameter and in life-threatening complications, such as aortic dissection or vessel rupture (Tsai et al., 2005). Depending on their etiology, TAAs are classified into two main forms: (1) sporadic TAA (sTAA), the most common and caused by the interplay between typical cardiovascular risk factors (e.g., hypertension, aging, and smoking) and unknown genetic alteration and/or de novo mutations, (2) familial TAA, which is genetically determined. Among familial forms, syndromic TAAs are worth mentioning, as they are associated with multi-organ disorders, such as vascular Ehlers-Danlos, Loeys-Dietz, and Marfan syndrome (MFS) (Balistreri, 2015). The global incidence of TAA is 5–10 per 100,000 persons per year, accounting for 150,000–200,000 deaths per year (Gouveia E Melo et al., 2022). Despite this burden, available therapies are merely palliative, and surgical replacement of the thoracic aorta with a prosthetic graft remains the only effective option (Papakonstantinou and Rorris, 2021; Perrucci et al., 2017). Therefore, the characterization of key molecular mechanisms leading to aortic dilation is necessary to identify relevant targets and develop innovative and etiological therapies (Bossone and Eagle, 2021). Despite the different etiologies, TAA share common pathological features, including excessive fibrotic deposition within the aortic wall driven by an overactivation of TGF-β signalling (Habashi et al., 2006; Holm et al., 2011; Takeda et al., 2018), as well as extracellular matrix (ECM) integrity disruption (Berger et al., 2025; El-Hamamsy and Yacoub, 2009). Dysfunctional ECM deposition and remodelling impair mechanotransduction, a process that critically regulates cellular phenotype and function in several pathological conditions. In particular, it has been demonstrated that modifications of physiological mechanosensing and mechanotransduction pathways in aortic vascular smooth muscle cells (VSMCs) may contribute to TAA pathogenesis and progression in the MFS context (Chaudhry et al., 2007; Humphrey and Tellides, 2019).

A key role in cellular mechanotransduction is played by integrins, heterodimeric transmembrane receptors composed of “α” and “β” subunits (Humphries, 2000). Integrins function as bidirectional mediators between the extracellular environment and the intracellular cytoskeleton, thereby regulating a wide range of cellular processes (Santoro et al., 2019). Among integrins, those containing the αV subunit have a strong affinity for proteins containing the arginine-glycine-aspartic acid (RGD) sequence (e.g., vitronectin, fibronectin, and fibrillin-1) (Barczyk et al., 2010). The most important extracellular function of αV integrins is the release and activation of TGF-β from its ECM-stored latent complex. The binding of integrins to TGF-β N-terminal latent associated protein, one of the ECM storage structures of TGF-β, allows the mechanical pulling of this complex by the cells and the subsequent release of TGF-β (Keski-Oja et al., 2004). Active TGF-β regulates both canonical (SMAD2/3) and noncanonical (ERK1/2) signalling pathways, exacerbating the pro-fibrotic response (Leask and Abraham, 2004). However, the complex and dimorphic role of TGF-β in the aneurysm context has been previously reported, since not only its upregulation but also its abrogation may lead to pathological worsening (Cook et al., 2015). The intracellular tail of αV integrins is strongly involved in different contexts in cytoskeletal organization and mechanotransduction pathway activation (e.g., small GTP-binding protein RhoA, its downstream effector ROCK1, and α-smooth muscle actin) (Costa et al., 2012; Ren et al., 1999; Schiller and Fässler, 2013). Both TGF-β and the mechanotransduction signalling pathway are key drivers of pro-fibrotic response in several cell types, tissues, and organs (Henderson et al., 2013; Tschumperlin et al., 2018). Previous studies, including ours, have reported that pharmacological inhibition of αV integrins in rat cardiac fibroblasts prevents their differentiation into myofibroblasts and production of pro-fibrotic molecules (Perrucci et al., 2018a) and attenuates fibrosis in a mouse model of cardiac fibrosis and systemic sclerosis (Bagnato et al., 2018; Sui and Hou, 2021). Enhanced expression of β3 integrin was reported in a murine model of MFS (*Fbn1*^C1039G/+^) (Parker et al., 2018), and αV integrin overexpression was detected in human iPSC-derived MFS-VSMC (Parker et al., 2018). Additionally, systemic *pan*-αV integrin inhibition by GLPG0187 reduced FAK-Akt-mTORC1 signalling, mitigated VSMC pro-fibrotic phenotypic switch, and limited aneurysm formation in *Fbn1*^C1039G/+^ mice (Nakamura et al., 2023). Altogether, these data confirm an important involvement of integrins, and in particular of αV integrin, in TAA. Among integrin inhibitors, there are several compounds able to mimic the RGD tripeptide, recognized by αV integrins. Despite their similar structure, the RGD-mimetic integrin antagonists show different selectivity. For example, GLPG0187 acts as a broad-spectrum integrin inhibitor with nanomolar activity against several RGD-binding integrins, whereas Cilengitide preferentially inhibits αVβ3 and αVβ5 integrins (Henderson and Sheppard, 2013). So, here we want to compare the effectiveness of integrin inhibitors in limiting progression of TAA in syndromic and sporadic forms of aneurysms and to determine whether a selective integrin inhibition may provide greater therapeutic benefits than global integrin blockade in all TAA contexts.

## Results

### αV integrin, ECM proteins, and pro-fibrotic mediator expression levels are higher in the thoracic aorta of patients with both MFS and sTAA than in healthy subjects

We performed spatial transcriptomics on samples of healthy control (HC) ascending aortas and on both dilated and non-dilated zones of ascending thoracic aortas obtained from patients affected by either MFS-TAA or sTAA, whose features are reported in Table S1 A.

**Figure 1.**
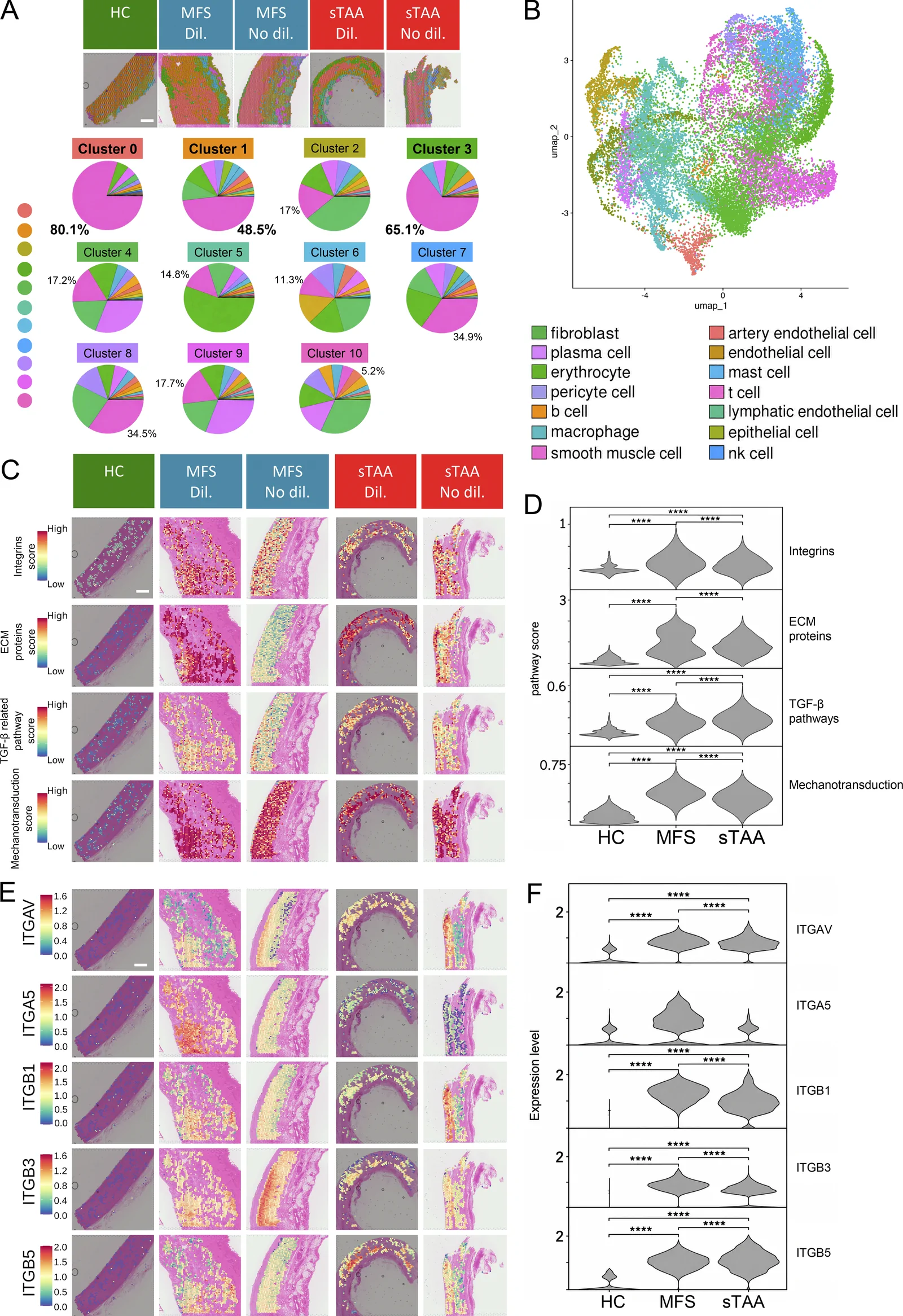
**Transcription levels of genes encoding ECM components, TGF-β signalling mediators, mechanotransduction proteins, and integrins are higher in TAA of MFS and non-syndromic (sTAA) patients than in HC aortas. (A, C, and E)** Spatial transcriptomics analysis on thoracic aortas from HC subjects and patients with MFS or sTAA, both in dilated (Dil.) and non-dilated (No dil.) aneurysmal regions. Scale bar = 1 mm. Aortic layers are displayed across all samples, from left to right, as intima, media, and adventitia. Images in A represent spatial spots. These spots have been detailed in pie charts, which summarize the aortic cell types defined by similar transcriptomic clusters. scRNA-seq data from the Tabula Sapiens vascular dataset (https://tabula-sapiens-portal.ds.czbiohub.org) have been used to perform the deconvolution and to attribute cellular composition to the spatial spots. The clusters highlighted (clusters 0, 1, and 3, in bold) contain the highest number of cells with transcriptional features attributable to VSMCs. **(B)** UMAP projection of the relative density and distribution of the 14 cellular macro-groups in the aortic wall. Images in C and D display the transcript number density (blue: low number, red: high number), respectively, of four different gene clusters and of five specific genes encoding integrins in spatial clusters with more than 45% VSMCs (0,1, and 3). **(D and F)** Violin plots of the gene expression levels on the basis of spatial density distribution. ****P < 0.0001.

**Figure S1.**
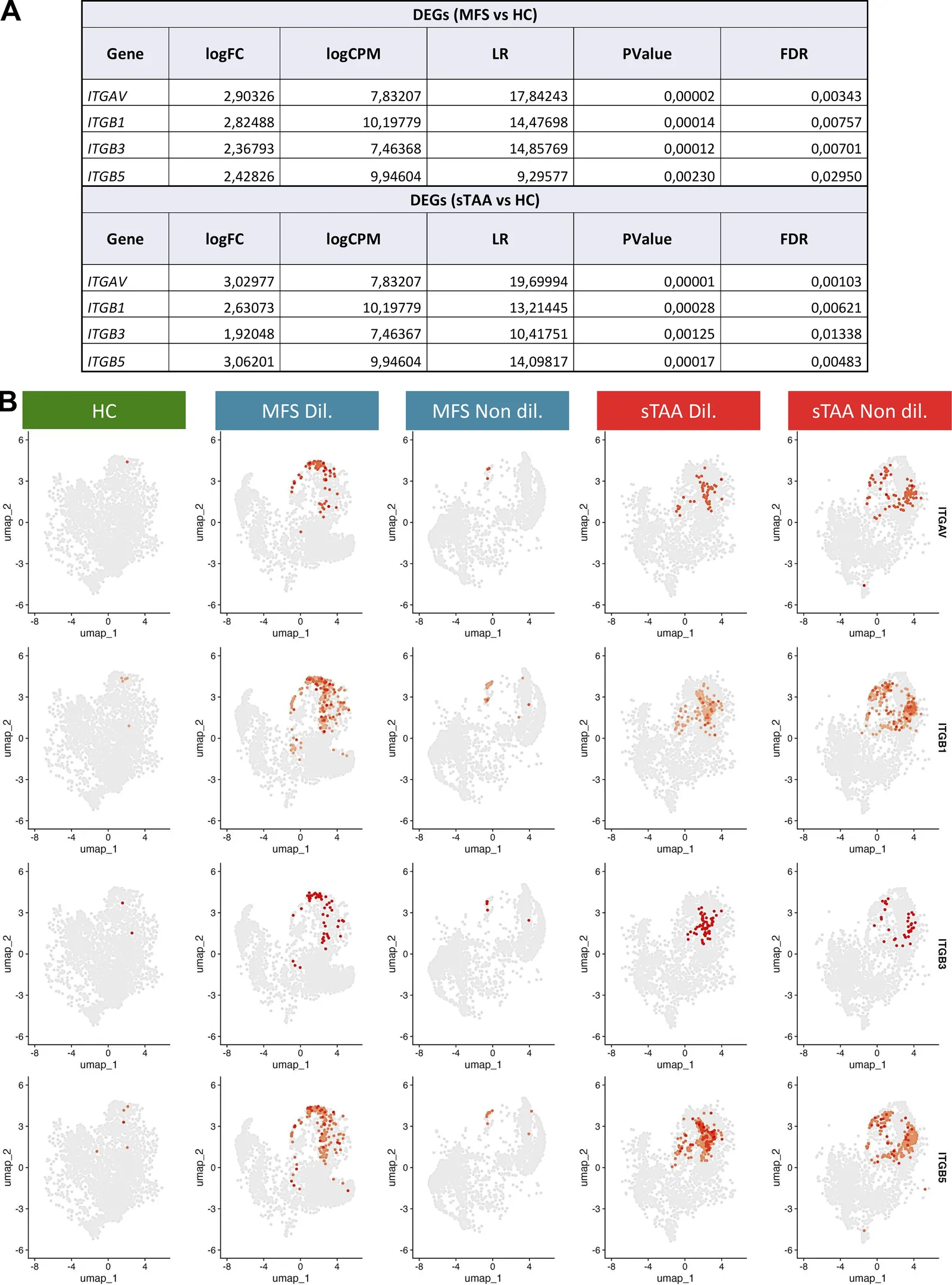
**Integrin-related genes are predominantly expressed in VSMCs from MFS and sTAA aortas** **. (A)** Tables reporting DEGs related to integrins obtained from spatial transcriptomics analysis data. **(B)** UMAP-based feature plots of the integrin genes (*ITGAV*, first row; *ITGB1*, second row; *ITGB3*, third row; *ITGB5*, fourth row). The gene expression normalized levels in VSMC are displayed with a shades-of-red color scale, from dark red (single-cell transform, SCT > 3.0, more highly expressed) to light red (SCT < 3.0, less expressed).

**Figure S2.**
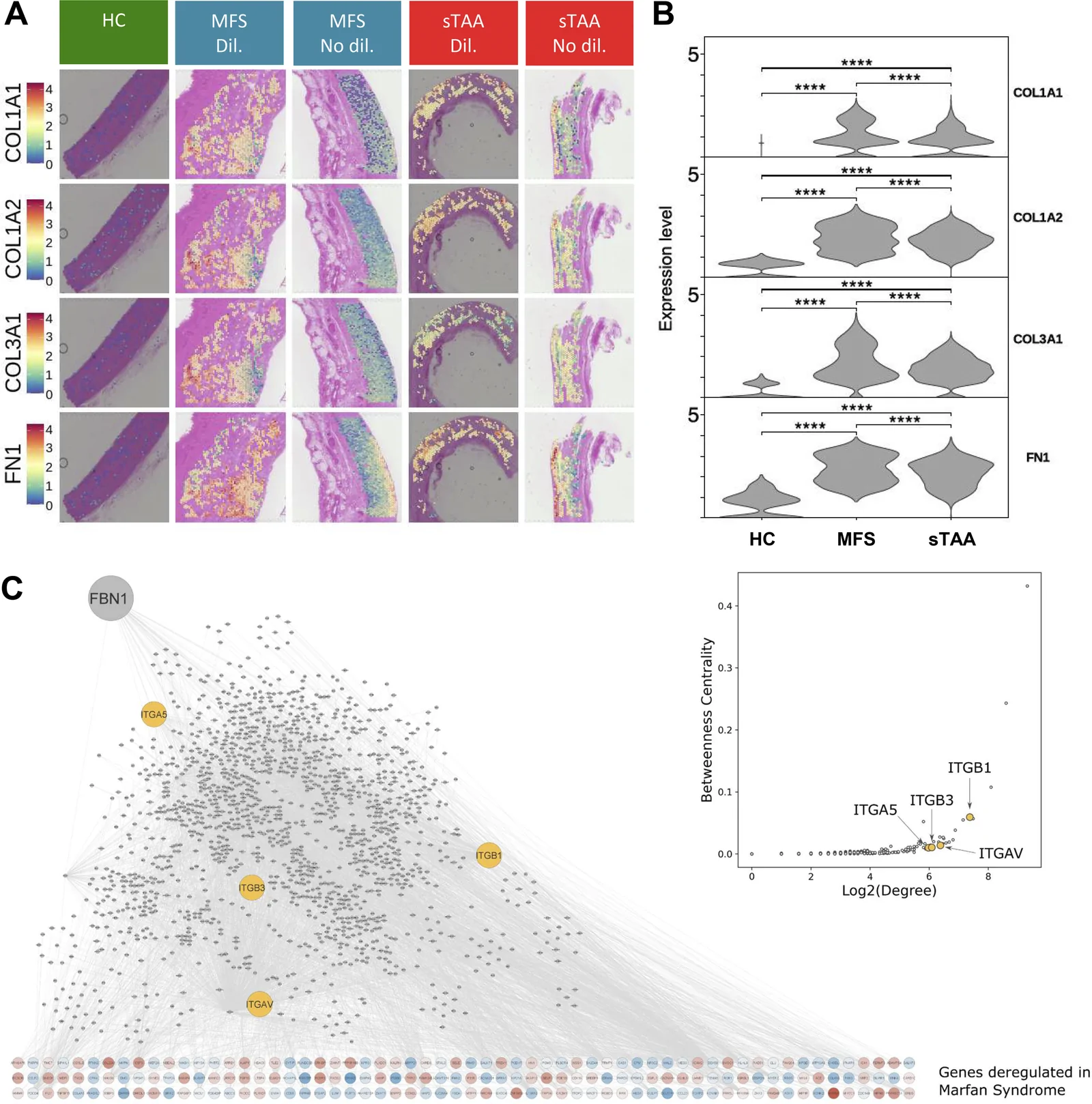
**Collagen-related genes are upregulated in MFS and sTAA aortas, and integrins emerge as central nodes in aneurysm pathogenesis** **. (A and B)** Spatial transcriptomics images (A) and relative violin plots (B), depicting the expression levels of genes encoding COL1A1 (first row), COL1A2 (second row), COL3A1 (third row), and fibronectin (FN1, fourth row) on thoracic aortas from HC subjects and patients with MFS or sTAA, both in dilated and non-dilated aneurysmal regions. The violin plots show the expression levels of gene groups in terms of spatial density distribution in cell clusters with more than 45% VSMCs. ****P < 0.0001. **(C)** Integrins play a key role in the protein-protein interaction (PPI) subnetwork relevant to MFS. This network connects FBN1 to genes deregulated in aortic smooth muscle cells from MFS patients through known PPIs. Genes upregulated in MFS are shown in red; downregulated genes in blue. Integrins (*ITGB1*, *ITGAV*, *ITGB3*, and *ITGA5*) are highlighted in yellow. The scatterplot in the insert represents the log-transformed degree and the betweenness centrality of all the nodes in the network.

**Figure 2.**
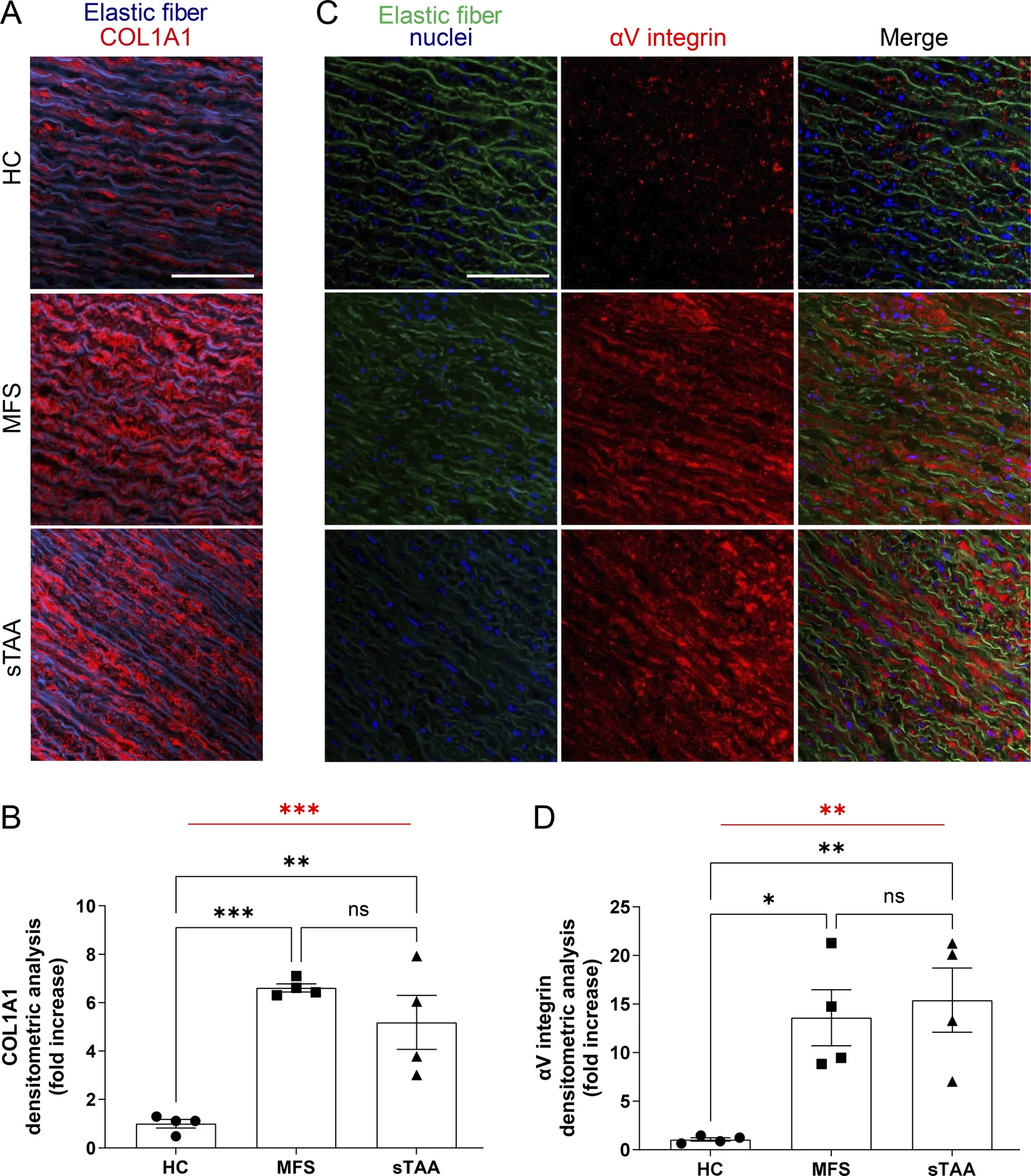
**COL1A1 and αV integrin are more abundant in tunica media of MFS-TAA and sTAA than in HC patients. (A and B)** Representative immunofluorescence images (A) and quantification (B) of COL1A1 expression in the tunica media of HC, MFS-TAA, and sTAA aortas. **(C and D)** Representative immunofluorescence images (C) and quantification (D) of αV integrin expression in the tunica media of HC aortas, MFS-TAA, and sTAA. Nuclei are stained with DRAQ5 and shown in blue. In all immunofluorescence images, the tunica intima and adventitia are not visible and are localized in the upper right and lower left corners, respectively. Scale bar = 100 μm. Data are shown as mean ± SEM, *n* = 4/group. One-way ANOVA (in red): **P < 0.01, ***P < 0.001. Tukey’s post hoc test (in black): *P < 0.05, **P < 0.01, and ***P < 0.001. Experimental data were verified in at least two independent experiments.

Data were processed in R, using the Seurat workflow (Hao et al., 2024), and spots capturing fewer than 200 genes were excluded from the analysis. Data from all samples were integrated and subjected to clustering to identify groups of spots with similar transcriptional profiles. This approach allowed segmentation of tissue sections into 11 spot clusters, with shared transcriptomic profiles across patients, as shown in Fig. 1 A. Specifically, clusters 0, 1, 3, 7, and 8 mapped to the medial layer, while the other clusters mapped to the intima and adventitia. To disentangle the cellular composition of the analyzed clusters, we interrogated the “vasculature” subset in the single-cell RNA Tabula Sapiens dataset (Tabula Sapiens Consortium et al., 2022), using the non-negative least squares (NNLS) method implemented in the R package semla (Larsson et al., 2023). The aortic cell type transcriptional profile is displayed in Fig. 1 B, and the average percentage of the cell type complexity within each spatial cluster is shown in Fig. 1 A pie charts. Next, we evaluated only clusters including more than 45% VSMCs (clusters 0, 1, and 3) and pathways known to be implicated in TAA, such as integrin signalling, ECM remodelling, TGF-β signalling, and mechanotransduction (Fig. 1, C and D). Genes involved in these pathways were significantly more highly expressed in sections from TAA patients, in both dilated and non-dilated regions, than in HC. Notably, the expression of integrins and mechanotransduction mediators was more prominent in the medial layer. Finally, differential gene expression analysis was performed by aggregating spot data into pseudo-bulk samples and comparing groups using edgeR. This analysis confirmed the upregulation of integrin genes, such as *ITGAV*, *ITGB1*, *ITGB3*, and *ITGB5* (Fig. 1, E and F; and Fig. S1 A), and collagen remodelling genes (Fig. S2, A and B; Table S1 B) in spot clusters enriched in VSMC for both TAA patients versus HC. As potentially expected, integrin genes were more highly expressed in the VSMC from dilated regions of aneurysm in comparison with non-dilated areas (Fig. S1 B). We validated the spatial transcriptomics data on collagen I (*COL1A1*) and *ITGAV* by assessing the protein expression levels of COL1A1 and αV integrin on human thoracic aortic tissues and found a significantly higher expression of both proteins in the tunica media of MFS-TAA and sTAA compared with HC, as shown in Fig. 2.

To investigate the central role of integrins in human TAA pathogenesis, we performed an in silico nonstandard system biology analysis leveraging public RNA-sequencing databases of aortic MFS-VSMC. First, we considered genes known to be deregulated in primary human MFS-VSMC, shown in red (upregulated) and blue (downregulated) in the bottom part of Fig. S2 C, as deposited by Hansen et al. (2019). Next, we built the protein–protein interaction network comprising high-confidence interactions from well-known public databases. To ensure tissue-specificity, we interrogated the Genotype-Tissue Expression database, which collects updated data on the expression and interactions of genes in specific anatomical niches, relative to the human thoracic aorta. This analysis suggests that integrins (in yellow) may work as hubs in the region of the network responsible for connecting fibrillin-1 to genes deregulated in MFS-VSMC. These data are further depicted in the graph in the inset, which clearly shows that integrins have a high degree of connections (log_2_ node degree) and a high betweenness centrality value, which measures how often a certain vertex lies on the shortest paths between other vertices, displaying a key role in the connectivity between fibrillin-1 and MFS-deregulated genes.

### Primary human VSMCs from thoracic aortic wall of TAA patients express high levels of αV integrins

**Figure S3.**
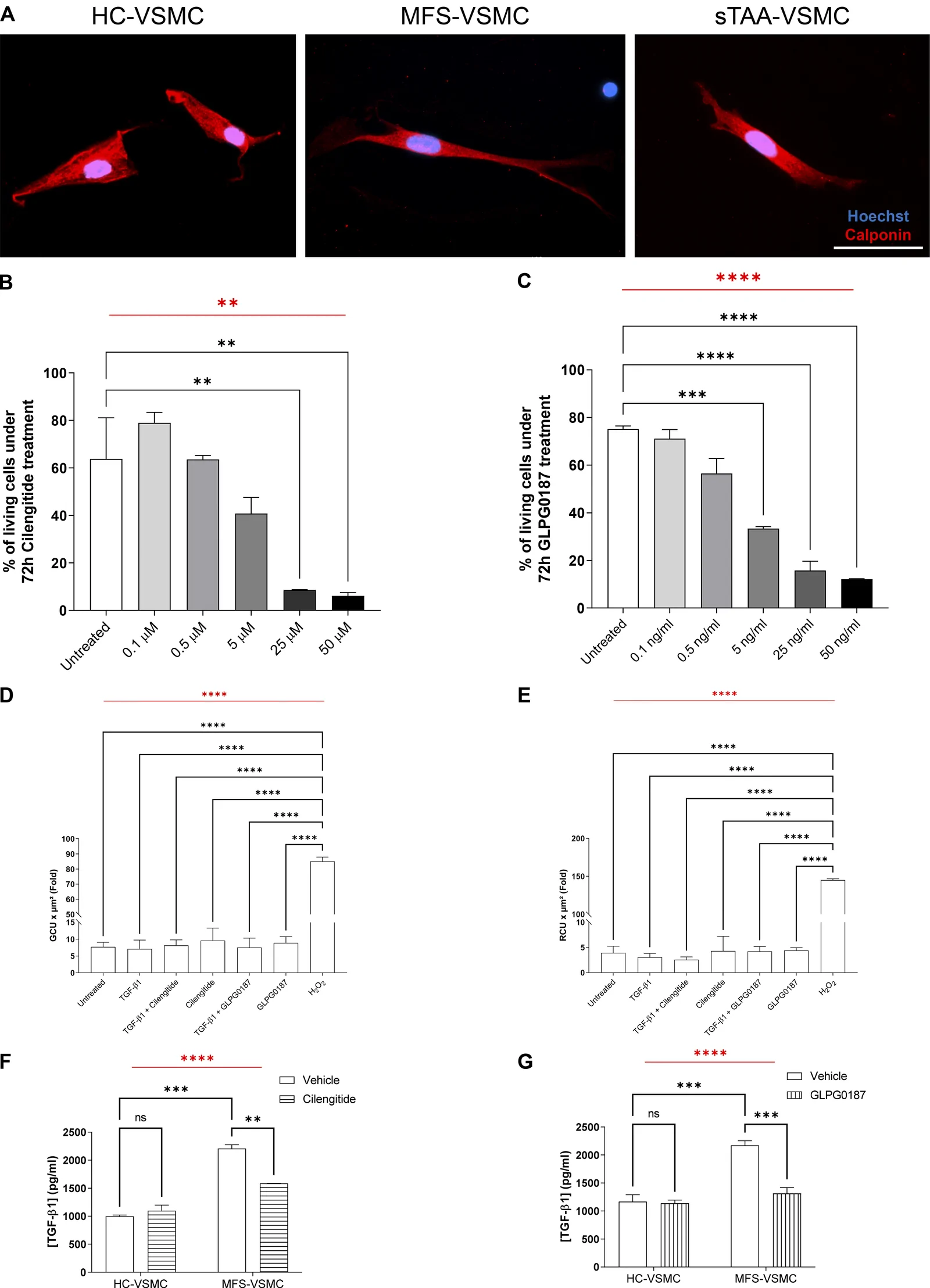
**Cilengitide and GLPG0187 reduce TGF-β1 release without inducing cytotoxicity in patient-derived VSMC.** **(A)** Immunofluorescence images of VSMC isolated from HC-, MFS-, and sTAA-aortas stained with calponin (in red). Nuclei have been stained with Hoechst (in blue). Magnification: 40×. Scale bar: 50 μm. **(B and C)** Dose-response MTT assay for Cilengitide (B) and GLPG0187 (C) on MFS-VSMC treated for 72 h. Data are shown as mean ± SD, *n* = 3/group. Two-way ANOVA (in red): **P < 0.001; ****P < 0.0001. Tukey post hoc test: **P < 0.001, ***P = 0.0001, ****P < 0.0001. **(D and E)** Pro-apoptotic and necrotic effects on MFS-VSMC of Cilengitide and GLPG0187 in vitro treatment for 48 h, by Annexin V-FITC (D) and EtBr (E) assays. H_2_O_2_ has been used as a positive control for cell death. Data are shown as mean ± SD, *n* = 6/group. Two-way ANOVA (in red): ****P < 0.0001. Tukey post hoc test: ****P < 0.0001. **(F and G)** Graph bars of ELISA assay results for TGF-β1 in supernatants of HC- and MFS-VSMC treated with vehicle (white bars), Cilengitide (F, striped bars), or GLPG0187 (G, striped bars). Data are shown as mean ± SD, *n* = 3/group. Two-way ANOVA (in red): ****P < 0.0001. Tukey post hoc test: **P < 0.01, ***P < 0.001.

**Figure 3.**
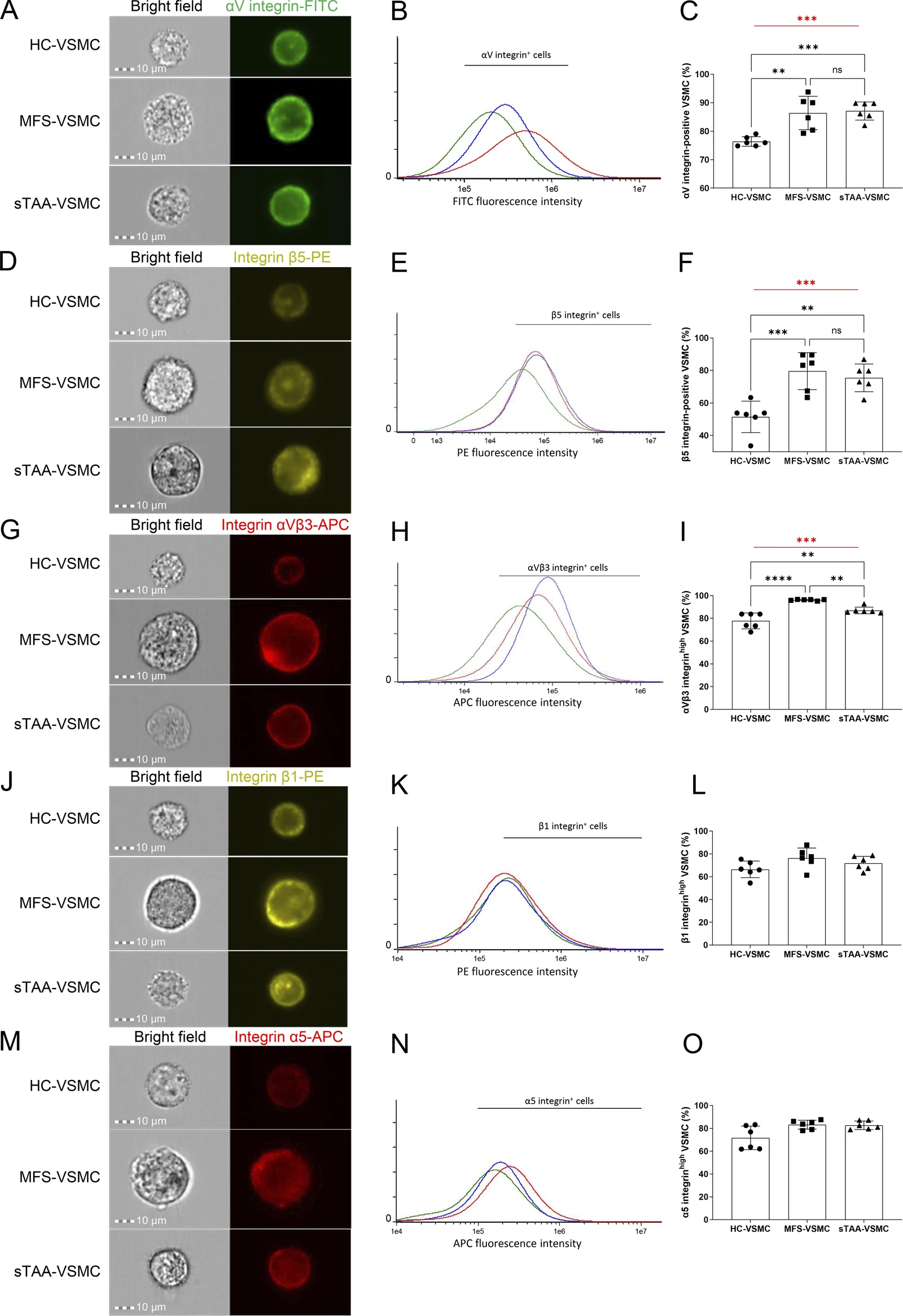
**αV, αVβ5, αVβ3, β1, and α5 integrins are more highly expressed in TAA-VSMC than in HC-VSMC. (A, D, G, J, and M)** ImageStreamX representative images for αV integrin (A, in green), β5 integrin (D, in yellow), αVβ3 integrin (G, in red), β1 integrin (J, in yellow), and α5 integrin (M, in red) staining in HC-, MFS-, and sTAA-VSMC. Cells are also shown in bright field (left images). Scale bars = 10 μm. **(B, C, E, F, H, I, K, L, N, and O)** Histograms representing the distribution of positive HC-VSMC (green line), MFS-VSMC (blue line), and sTAA-VSMC (red line) for each integrin. Bar graphs represent the percentage of gated cells positive for αV integrin (B and C), β5 integrin (E and F), αVβ3 integrin (H and I), β1 integrin (K and L), and α5 integrin (N and O). Data are expressed as mean ± SD, *n* = 6/group. One-way ANOVA (in red): *P < 0.05, ***P = 0.0001. Tukey’s post hoc test (in black): **P < 0.001, ***P = 0.0001, and ****P *<* 0.0001. Experimental data were verified in at least two independent experiments.

We isolated VSMCs from MFS and sTAA thoracic aortic wall and compared them with HC-VSMCs. We first confirmed the identity of the isolated cells by staining them for the VSMC marker calponin (Fig. S3 A). Next, we evaluated the expression levels of RGD-binding integrins that should be present in the aortic wall (i.e., αV, β5, αVβ3, β1, and α5 integrins) on the VSMC surface, using the ImageStreamX technology, which combines flow cytometry with cell imaging. The percentage of VSMC expressing αV (Fig. 3, A–C), β5 (Fig. 3, D and F), and αVβ3 (Fig. 3, G–I) was greater in both MFS- and sTAA-VSMC than in HC-VSMC. In contrast, β1 and α5 integrin expression did not vary between groups (Fig. 3, J–O), pinpointing the potential role of αVβ3 and αVβ5 among all the RGD-binding integrins. Thus, primary VSMCs recapitulate the pathological increase in integrin expression previously documented in the transcriptome of aortic tissues from TAA patients.

### αV integrin inhibition downregulates pro-fibrotic molecular signatures in MFS- and sTAA-VSMCs

On the basis of the data on expression levels of integrins, we wanted to explore whether the selective inhibition of αVβ3 and αVβ5 dimers by Cilengitide was effective in reducing pro-fibrotic molecular signatures in cultured VSMCs and compare its efficacy with the *pan*-αV integrin inhibitor GLPG0187.

First, we studied the toxicity of the two compounds by exposing primary HC-VSMCs to multiple doses and measuring cell viability at 72 h (Fig. S3, B and C). We chose the concentration of 0.5 µM for Cilengitide and the concentration of 0.5 ng/ml for GLPG0187 for subsequent in vitro experiments. We also validated the safety of the selected doses, either alone or in combination with TGF-β1 (to mimic pro-fibrotic conditions) on living VSMCs. We did not observe significant differences in the number of Annexin V^+^ apoptotic cells (Fig. S3 D) or necrotic cells (Fig. S3 E) in any of the tested conditions.

Since αV integrins are known to release the active form of TGF-β1 from its latent complex, we evaluated the effectiveness of both Cilengitide and GLPG0187 in cultured HC- and MFS-VSMCs by measuring TGF-β1 release. Fig. S3, F and G show the significant reduction in the concentration of TGF-β1 released in VSMC supernatant, particularly in MFS patient–derived cells, upon treatment with both αV integrin inhibitors.

**Figure S4.**
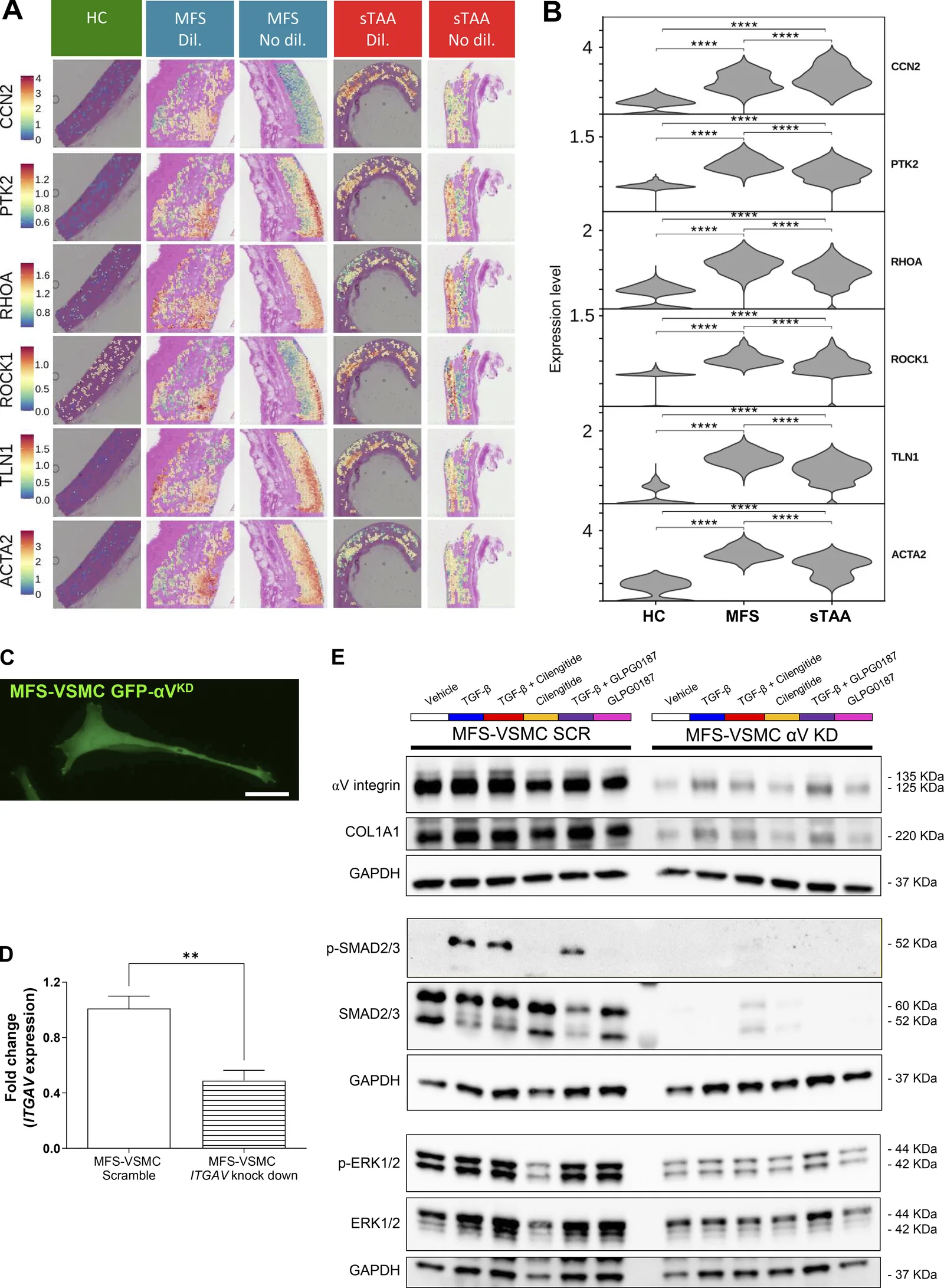
**αV integrin regulates mechanotransduction and TGF-β–related profibrotic signaling in MFS and sTAA VSMCs** **. (A and B)** Spatial transcriptomics images (A) and relative violin plots (B), depicting the expression levels of genes encoding CCN2 (first row), PTK2 (second row), RHOA (third row), ROCK1 (fourth row), TLN1 (fifth row), and ACTA2 (sixth row) on thoracic aortas from HC subjects and patients with MFS or sTAA, both in dilated and non-dilated aneurysmal regions. The violin plots show the expression levels of gene groups in terms of spatial density distribution in cell clusters with more than 45% VSMCs. ****P < 0.0001. **(C)** Representative image of MFS-VSMC knocked down for ITGAV (αVKD GFP-positive cell, in green) by lentiviral particle infection. Magnification = 40×. Scale bar = 100 μm. **(D)** Real-time PCR assay quantification for ITGAV in MFS-VSMC infected with lentiviral particles. Graph bars represent fold increase of ITGAV gene in total cDNA obtained by cell after infection with scramble sequence (white bar) or with shRNA sequence specific for ITGAV (striped bar). Data are shown as mean ± SD, *n* = 4/group. Student’s *t* test: **P < 0.001. **(E)** Western blot images for αV integrin, COL1A1, total and phosphorylated SMAD2/3, and total and phosphorylated ERK1/2 on total protein cell extract from MFS-VSMC infected with scramble shRNA (MFS-VSMC SCR) and αVKD (MFS-VSMC αV KD). Infected MFS-VSMCs were treated with vehicle (white bars), TGF-β1 (blue bars), TGF-β1 + Cilengitide (red bars), Cilengitide only (orange bars), TGF-β1 + GLPG0187 (purple bars), and GLPG0187 only (pink bars). GAPDH has been used as a protein loading control. Source data are available for this figure: SourceData FS4.

**Figure 4.**
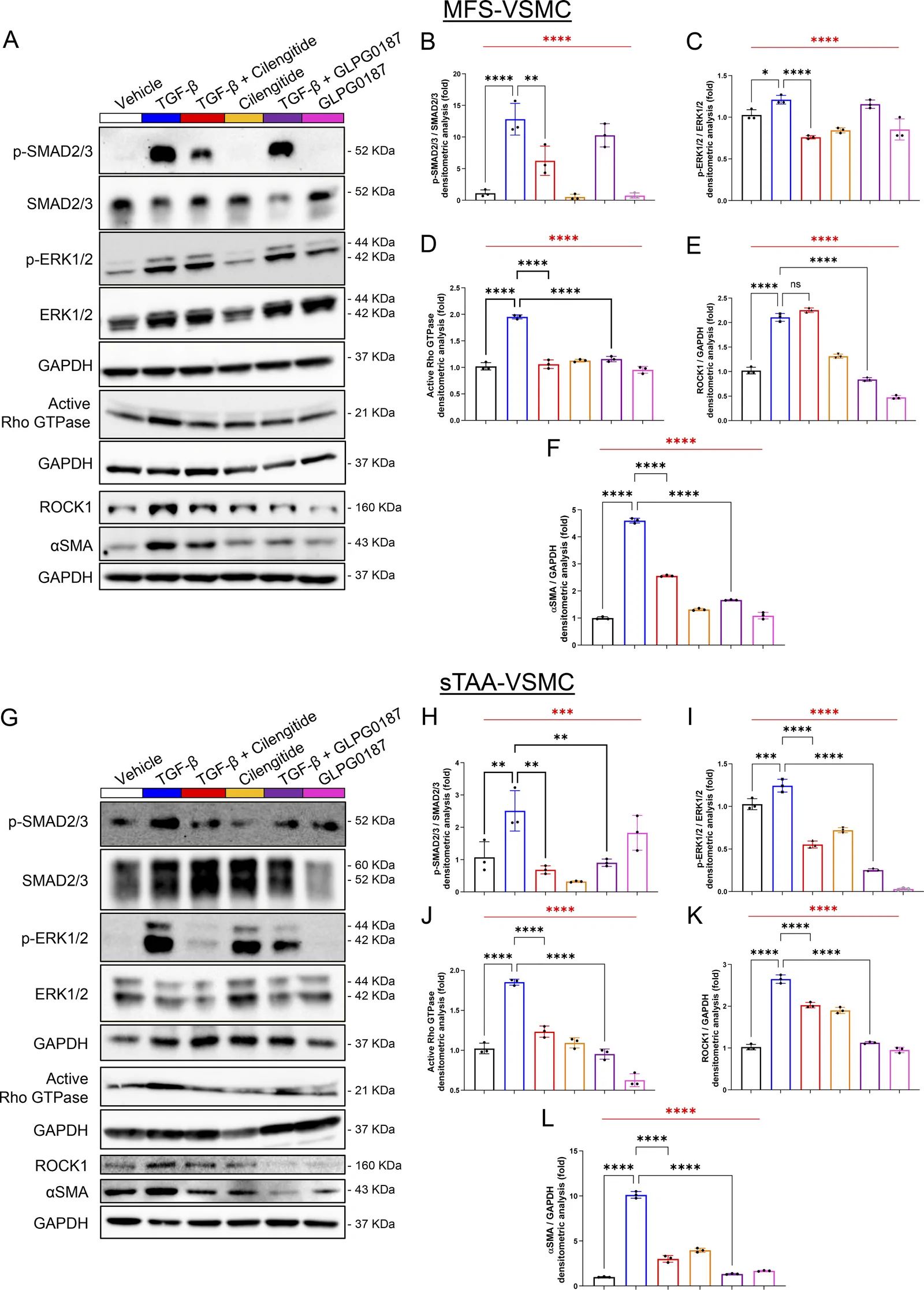
**αV integrin inhibition inhibits TGF-β signalling and mechanotransduction pathways in both MFS- and sTAA-VSMC. (A)** Representative western blots for the proteins of interest in MFS-VSMC in response to the indicated treatments. **(B–F)** Densitometric analyses for total:phosphorylated SMAD2/3 ratio (B), total:phosphorylated ERK1/2 ratio (C), active RhoA GTPase (D), ROCK1 (E), and αSMA (F). **(G)** Representative western blots for the proteins of interest in sTAA-VSMC in response to the indicated treatments. **(H–L)** Densitometric analyses for total:phosphorylated SMAD2/3 ratio (H), total:phosphorylated ERK1/2 ratio (I), active RhoA GTPase (J), ROCK1 (K), and αSMA (L). GAPDH has been used to normalize RhoA GTPase, ROCK1, and αSMA expression. Data are shown as mean ± SD, *n* = 3/group. One-way ANOVA (in red): ****P *<* 0.0001. Tukey’s post hoc test (in black): *P < 0.05, **P < 0.01, ***P = 0.0001, and ****P < 0.0001. Experimental data were verified in at least two independent experiments. Source data are available for this figure: SourceData F4.

Our spatial transcriptomic data indicated that several mediators of pro-fibrotic and mechanotransduction pathways were significantly upregulated in both MFS and sTAA aortic tissues (Fig. S4, A and B; and Table S1 B). Thus, we focused on the effect of integrin inhibitors in patient-derived VSMCs cultured with or without human recombinant TGF-β1. Fig. 4 shows representative western blot images and quantification of activation of both canonical (SMAD2/3) and noncanonical (ERK1/2) pro-fibrotic TGF-β1 signalling pathways, as well as of integrins downstream mechanotransduction mediators (i.e., RhoA GTPase, ROCK1, and αSMA) in total protein extracts of MFS-VSMC (Fig. 4, A–F) and sTAA-VSMC (Fig. 4, G–N). As expected, treatment with TGF-β1 significantly increased the activation of these pathways in both cell types. Integrin inhibitors effectively limited the activation of TGF-β1 and mechanotransduction pathways in sTAA-VSMC, whereas Cilengitide was the only drug able to significantly decrease the activation of canonical and noncanonical TGF-β1 signalling pathways in MFS-VSMC. Mechanotransduction was similarly inhibited by both drugs in MFS-VSMC, except for ROCK1 expression levels, which were not modified by Cilengitide. Minor differences were observed in response to both treatments in the absence of TGF-β1.

Thus, in the pro-fibrotic context, the αV integrin inhibitors potently limit the activation of both TGF-β1 and mechanotransduction pathways in primary VSMCs. Specifically, Cilengitide is more effective than GLG0187 in limiting TGF-β1 activation in both TAA cell types.

### αV integrin inhibition downregulates COL1A1 expression levels in MFS- and sTAA-VSMCs and Fb

**Figure 5.**
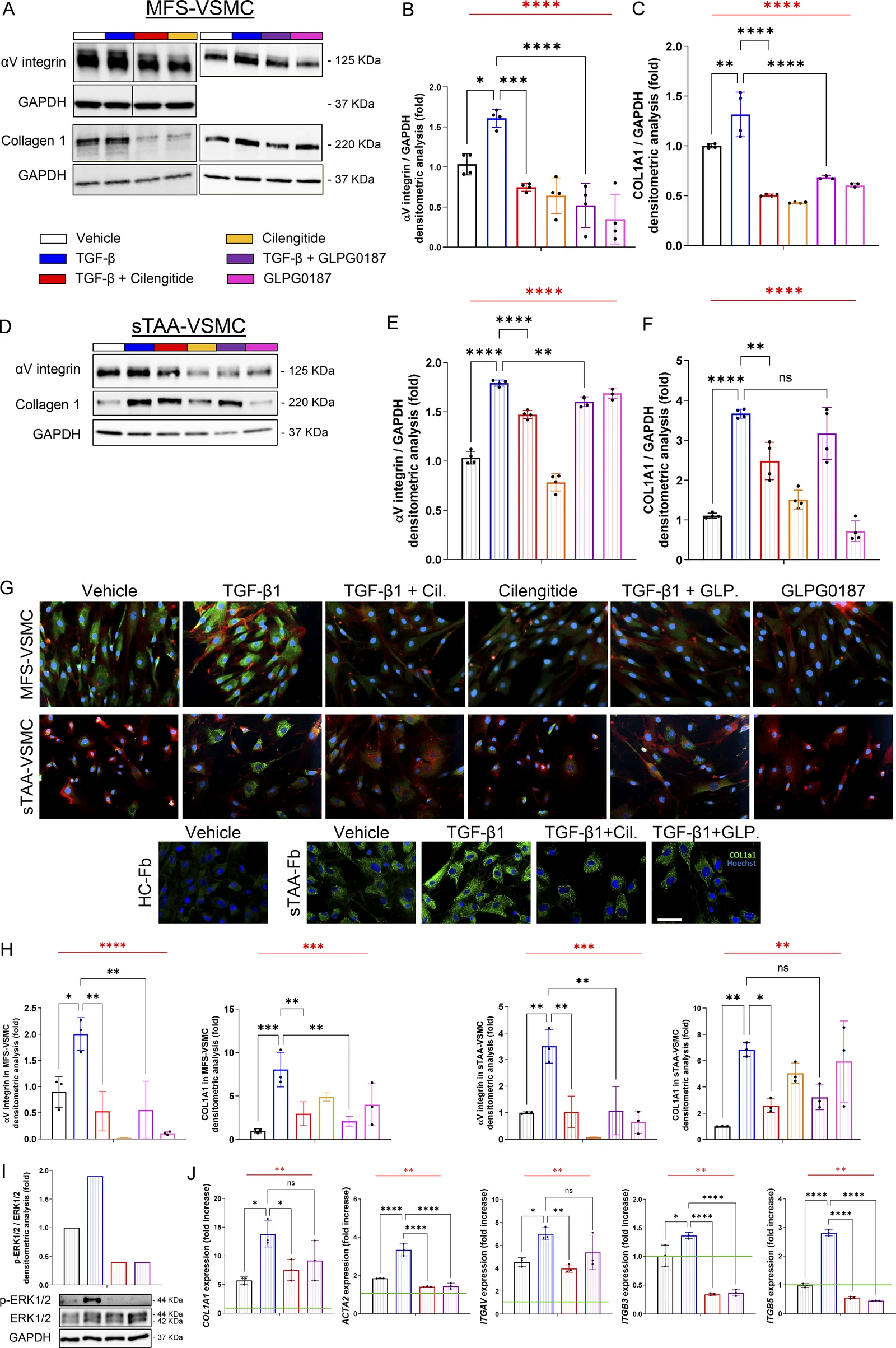
**Inhibition of αV integrin downregulates the expression levels of αV integrin and COL1A1 in both MFS- and sTAA-VSMCs. (A–C)** Representative western blot images (A) and corresponding densitometric analysis quantification for αV integrin (B) and COL1A1 (C) in MFS-VSMCs exposed to the indicated treatments. **(D–F)** Representative western blot images (D) and corresponding densitometric analysis quantification for αV integrin (E) and COL1A1 (F) in sTAA-VSMCs exposed to the indicated treatments. GAPDH has been used as a protein loading control. Data are shown as mean ± SD, *n* = 4/group. One-way ANOVA (in red): ****P < 0.0001. Tukey’s multiple comparisons post hoc test: *P < 0.05; **P < 0.01; ***P = 0.0001; ****P < 0.0001. **(G and H)** Representative images (G) and corresponding quantifications (H) of immunofluorescence staining for αV integrin and collagen I (COL1A1) in MFS- and sTAA-VSMC and Fb exposed to the indicated treatments. Nuclei were stained with Hoechst and are shown in blue. Scale bar = 50 μm. Data are shown as mean ± SD, *n* = 3/group. One-way ANOVA (in red): ***P = 0.0001; ****P < 0.0001. Tukey’s multiple comparisons post hoc test: *P < 0.05; **P < 0.01; ***P = 0.0001. **(I)** Western blot images and relative densitometric analysis for total and phosphorylated ERK1/2 on sTAA-Fb exposed to the indicated treatments. GAPDH has been used as a protein loading control. *n* = 1/group. **(J)** COL1A1, ACTA2, ITGAV, ITGB3, and ITGB5 gene expression levels in RNA extracts of HC-Fb (green line) and sTAA-Fb after indicated treatments. qRT-PCR analyses were performed three times in triplicate, and data are shown as fold change ± SD, *n* = 3. One-way ANOVA (in red): **P < 0.01. Tukey’s post hoc test (in black): *P < 0.05, **P < 0.01, ***P = 0.0001, and ****P < 0.0001. Experimental data were verified in at least two independent experiments. Source data are available for this figure: SourceData F5.

Next, we investigated the effect of Cilengitide and GLPG0187 on the expression levels of COL1A1, one of the major ECM components in spatial transcriptomic data of TAA patients in Fig. 1. Moreover, since negative feedback loops often result in the downregulation of a receptor in the presence of its specific ligand, we also evaluated the expression levels of αV integrin by VSMC after treatment with its inhibitors. Fig. 5 shows the expression levels of αV integrin and COL1A1 in MFS-VSMC (Fig. 5, A–C, G, and H) and sTAA-VSMC (Fig. 5, D–H) as well as in sTAA-Fb (Fig. 5, G, I, and J). As expected, the presence of TGF-β1 significantly increased the expression of αV integrin and COL1A1 in patients’ cells when compared with vehicle. Cilengitide significantly downregulated the expression levels of αV integrin and COL1A1 in all cell types, while GLPG0187 was not able to limit COL1A1 expression in sTAA-VSMC and both COL1A1 and ITGAV gene expression levels in sTAA-Fb (Fig. 5 J).

These results were further confirmed in primary MFS patient–derived VSMC silenced for the ITGAV gene. In efficiently transduced EGFP^+^ VSMC (αV^KD^, Fig. S4 C), levels of ITGAV gene expression (Fig. S4 D), as well as αV integrin, COL1A1, and TGF-β1 canonical and noncanonical pathway mediators, were reduced when compared with scramble. Treatment with TGF-β1 in αV^KD^ cells resulted in minimal activation of TGF-β1 pathways and integrin and COL1A1 expression levels (Fig. S4 E).

Thus, αV integrin mediates the activation of TGF-β1 pathways and the production of the pro-fibrotic driver COL1A1. Cilengitide stands as the more effective drug in inhibiting αV integrin and, thereby, the activation of pro-fibrotic pathways in VSMCs of all TAA types.

### Cilengitide limits aneurysm progression in both syndromic and non-syndromic TAA murine models

Finally, we tested and compared the therapeutic potential of the two integrin inhibitors in two murine models of TAA, namely Fbn1^C1039G/+^ mice that recapitulate syndromic MFS-TAA and a model of sTAA, in which lysyl oxidase (LOX) is inhibited by daily administration of β-aminopropionitrile (BAPN) in drinking water for 3 wk from the sixth to the ninth wk of age in C57Bl/6 mice (Zheng et al., 2020). In the syndromic model, we treated both WT and MFS mice three times a week with either Cilengitide or GLP0187 from the 5th to the 16th wk of age. In the sTAA model, Cilengitide and GLP0187 were administered three times a week, at the same time as BAPN administration.

**Figure 6.**
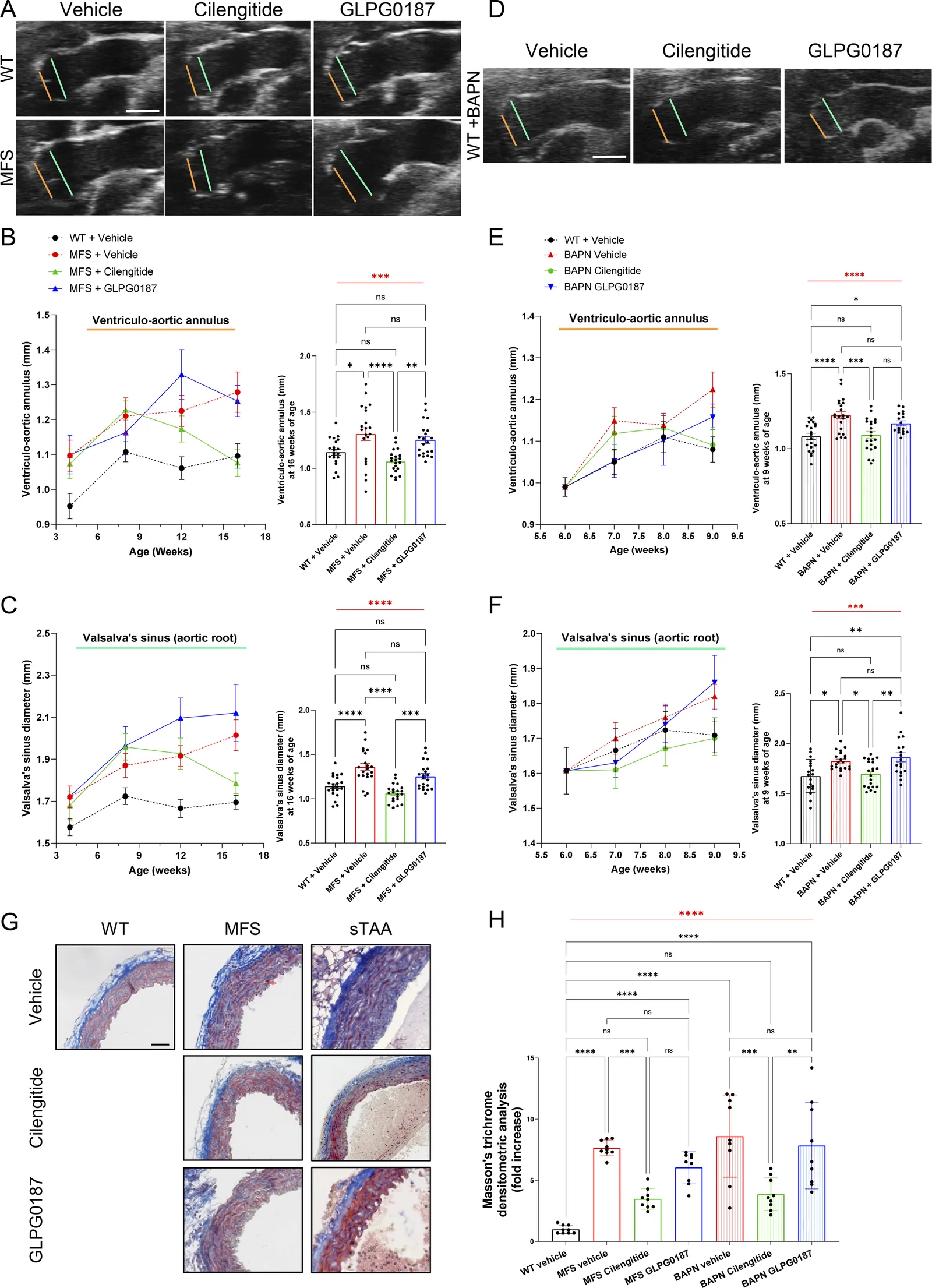
**Cilengitide limits thoracic aortic dilatation in both MFS and sTAA murine models. (A)** Representative 2D echocardiographic images of ventriculo-aortic annulus (orange line) and Valsalva’s sinus (turquoise line) diameters in WT and Fbn1^C1039G/+^ (MFS) mice, treated with vehicle, Cilengitide, or GLPG0187. **(B and C)** Quantification of ventriculo-aortic annulus (B) and Valsalva’s sinus (C) diameters in WT and MFS mice at the indicated time points. Graphs on the right show individual measurements at 11 wk, in triplicate. *n* = 12 (WT + vehicle); *n* = 13 (MFS + vehicle); *n* = 10 (MFS + Cilengitide); *n* = 11 (MFS + GLPG0187). **(D)** Representative 2D echocardiographic images of ventriculo-aortic annulus (orange line) and Valsalva’s sinus (turquoise line) diameters in mice treated with BAPN in combination with vehicle, Cilengitide, or GLPG0187. **(E and F)** Quantification of ventriculo-aortic annulus (E) and Valsalva’s sinus (F) diameters in BAPN-treated mice in combination with vehicle, Cilengitide, or GLPG0187 at the indicated time points. Graphs on the right show individual measurements at 2 wk of treatment, in triplicate. *n* = 7 (WT + vehicle); *n* = 7 (BAPN + vehicle); *n* = 7 (BAPN + Cilengitide); *n* = 6 (BAPN + GLPG0187). All 2D echocardiographic data are shown as mean ± SEM. One-way ANOVA (in red) with Tukey’s post hoc test for multiple comparisons (in black): *P < 0.05, **P < 0.01, ***P < 0.001, and ****P < 0.0001. **(G and H)** Representative images of Masson’s trichrome staining (G) and relative quantification (H) of collagen (blue signal) in the tunica media of thoracic aortas from WT, MFS, or BAPN mice, treated with vehicle, Cilengitide, or GLPG0187. Data are shown as mean ± SD, *n* = 9. One-way ANOVA (in red) with Tukey’s multiple comparisons post hoc test (in black): *P < 0.05, **P < 0.01, ***P < 0.001, and ****P < 0.0001. Experimental data were verified in at least two independent experiments.

By 2D echocardiographic analysis, we monitored the size of two anatomical locations of the ascending aorta, namely the ventriculo-aortic annulus and the Valsalva’s sinus, shown in orange and turquoise in Fig. 6, respectively. As expected, MFS mice showed statistically higher values for both annulus and Valsalva’s sinus diameters than WT mice at all time points. As shown in Fig. 6, B and C, Cilengitide effectively limited aortic dilation at both locations at 16 wk, whereas GLPG0187 did not show any statistical difference from MFS mice treated with vehicle. Mice treated with BAPN for 3 wk (from the sixth to the ninth wk of age) showed an aortic dilation comparable with that observed in MFS mice at the 16th wk of age, confirming the robustness of the model. Also in this case, Cilengitide was the only effective drug in limiting aortic dilation at both locations (Fig. 6, E and F).

**Figure 7.**
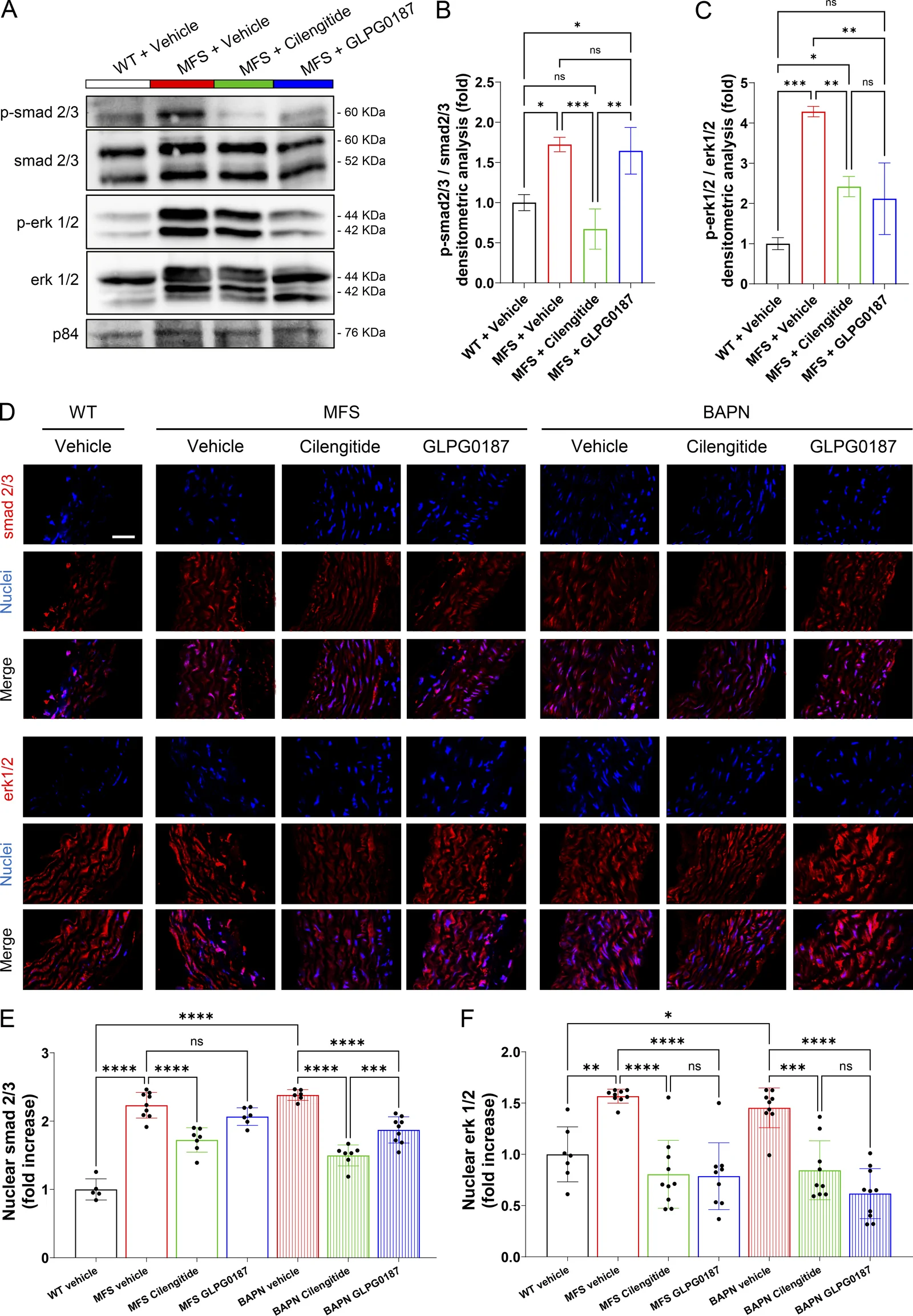
**Cilengitide limits thoracic aortic pro-fibrotic molecular mechanisms in both MFS and sTAA murine models. (A–C)** Western blot images (A) and their relative densitometric analyses for total and phosphorylated smad2/3 (B) and total and phosphorylated erk1/2 (C) on protein extracts from WT (black bars) and MFS thoracic aortic tissues, after treatments with vehicle (red bars), Cilengitide (green bars), and GLPG0187 (blue bars). p84 has been used as a protein loading control. Data are shown as mean ± SD, *n* = 3/group. One-way ANOVA and Tukey’s multiple comparisons post-test: *P < 0.05; **P < 0.01; ***P = 0.0001. **(D–F)** Immunofluorescence assay (D) and relative nuclear signal quantification of smad 2/3 (E) and erk 1/2 (F) on thoracic aortas of WT mice treated with vehicle (black bars, first column), MFS mice treated with vehicle (empty red bar, second column), Cilengitide (empty green bar, third column), GLPG0187 (empty blue bar, fourth column), and BAPN mice treated with vehicle (striped red bar, fifth column), Cilengitide (striped green bar, sixth column), and GLPG0187 (striped blue bar, seventh column). Nuclei were stained with DRAQ5 and are depicted in blue. Scale bar = 30 μm. Data are shown as mean ± SD, *n* = 3/group in triplicate. One-way ANOVA with Tukey’s post hoc test (in black): *P < 0.05, **P < 0.01, ***P < 0.001, and ****P < 0.0001. Experimental data were verified in at least two independent experiments. Source data are available for this figure: SourceData F7.

To evaluate collagen deposition in the aortic wall, we measured the extent of fibrosis in terms of processes and biochemical events in the media layer of thoracic aortas isolated from both MFS and sTAA murine models. Specifically, we performed Masson’s trichrome staining (Fig. 6, G and H) and protein expression level assays (Fig. 7 A) to evaluate the phosphorylation and the subsequent nuclear translocation of smad2/3 and erk1/2 transcription factors (Fig. 7 D). These analyses showed reduced collagen deposition in the aorta of both syndromic and non-syndromic TAA mouse models after treatment with Cilengitide, but not with GLPG0187, in comparison with vehicle-treated animals, confirming the echocardiography results. The activation of smad2/3 in MFS mice and MFS-VSMC showed a similar behavior; thus, treatment with Cilengitide, but not with GLPG0187, allowed the phosphorylation and the translocation of this transcription factor into the VSMC nuclei. On the contrary, in the sporadic context, both inhibitors are able in activating smad2/3 pathway. Concerning the noncanonical TGF-β pathway, activation and translocation of erk1/2 transcription factor were stimulated by both integrin inhibitors in all pathological contexts (Fig. 7, E and F).

Taken together the Fig. 6 and Fig. S4 results, we demonstrated that Cilengitide limits pro-fibrotic events and collagen deposition in both syndromic and sTAA models more efficiently than GLPG0187.

## Discussion

The results described in this manuscript provide novel fundamental insights into the understanding of TAA. We provided the first human thoracic aortic spatial transcriptomic dataset from healthy subjects, MFS-TAA, and sTAA patients (in both dilated and non-dilated regions). Then, the identified molecular signatures were validated in primary VSMC cultures obtained from multiple patients. These primary cells are far more representative of the human condition compared with iPSC-derived VSMCs used in previous studies (Nakamura et al., 2023). Lastly, we provide proof of concept on the therapeutic potential of Cilengitide, an orphan drug that appears more effective in limiting aortic dilation in both syndromic and non-syndromic TAA conditions than systemic *pan*-αV inhibitor GLPG0187. Collectively, our data confirm the central involvement of αVβ3 and αVβ5 integrins in TAA onset/progression and the effectiveness of their selective inhibition in mitigating the disease phenotype in both syndromic and non-syndromic aneurysms.

The possibility of interfering with integrin activity is becoming a central topic in the management of aortopathies. Recent evidence showed αV integrin overexpression in iPSC-derived aortic VSMCs from MFS-TAA patients, proposing the pathway mediated by mTORC as one of the key molecular mechanisms limiting MFS-TAA upon integrin inhibition with GLPG0187 (Nakamura et al., 2023). Here, we provide evidence for the relevance of additional signalling pathways, such as TGF-β and mechanotransduction, which may be selectively and effectively inhibited by Cilengitide, not only in MFS but also in non-syndromic TAA. This aspect poses a higher health and societal burden worldwide due to the enormous frequency of sTAA.

We and others have contributed to dissecting the role of integrins and associated mechanotransduction pathways in other cardiovascular diseases, mainly in hypertensive-derived cardiac fibrosis (Perrucci et al., 2018a; Perrucci et al., 2018b; Ross, 2004; Sarrazy et al., 2014). This led us to apply our findings to TAA (Perrucci et al., 2020), for which evidence from other scientists pointed to a relevant role of integrins (Humphrey et al., 2015; Parker et al., 2018; Pedroza et al., 2020a). The mediators analyzed in Fig. 5 were selected based on their documented involvement in pro-fibrotic mechanisms regulated by both TGF-β signalling and mechanotransduction (Humphrey et al., 2015). This initial choice was then further supported by their increased expression levels observed in TAA patient–derived specimens from spatial transcriptomics datasets (Fig. S3, A and B; and Table S1 B). The data presented in this study, indeed, provided the first characterization of human thoracic aortic specimens and also allowed some previous data confirmation or denial. For example, the expression levels of α5 integrin were surprisingly not statistically higher in VSMC of patients’ aortas, albeit a member of the RGD receptors and a target of integrin inhibitors (Cirkel et al., 2016). A recent article reported its involvement in TAA formation by in vivo experiments on the *Fbn1*^mgR/mgR^ mouse model (Chen et al., 2023). However, it is well known that the disease manifestations of this model are more severe -and often far from the real human pathological context-than in *Fbn1*^C1039G/+^ mice and also in MFS patients.

Our in vitro results suggested that TAA-VSMCs undergo a switch from a contractile to a secretory phenotype, and our spatial transcriptomics data solve an open question in the Nakamura et al. paper concerning the localization of the VSMC cluster expressing higher levels of integrins (Nakamura et al., 2023). The images shown in Fig. 1 strongly highlight that, among the cell clusters with a higher percentage of VSMC, the strongest gene expression of integrins (in general) and ITGAV is observed at the interface of the tunica media and adventitia of patients’ samples, specifically in the dilated region of both syndromic and non-syndromic aneurysms. This result paves the way for further studies aimed at characterizing this unique niche and the behavior of its cells.

We furthermore indicated in this study that the phenotypic switch of VSMC can be effectively reverted by αV integrin inhibition. This issue is debated in the TAA field (Hu et al., 2023; Lu et al., 2021; Mao et al., 2015; Pedroza et al., 2020b) with conflicting results. For example, Pan and colleagues have recently showed that αVβ3 integrin inhibition, mediated by macrophages-derived legumain, leads to Rho GTPase inactivation, which in turn suppresses VSMC phenotypic switch and that this exacerbates thoracic aortic dissection (Pan et al., 2022). On the contrary, our results demonstrate that αV integrin inhibition limits the switch of patient-derived VSMC into a secretory phenotype and downregulates fibrosis, protecting the thoracic aorta from dilation both in MFS and BAPN-treated animals. Surely, it will be interesting—and necessary—to evaluate by single-cell RNA sequencing (scRNA-seq) analysis in in vivo models the effects of integrin inhibition on this VSMC sub-cluster both in the syndromic and in the sporadic context.

Data shown here are well aligned with recent evidence on critical αV integrin involvement in TAA pathophysiology. The “traction model” (Keski-Oja et al., 2004), by which αV integrins bind ECM proteins and activate TGF-β from its latent form in the ECM, fits with the collected results, suggesting the role of Cilengitide in mitigating the detrimental mechanotransduction. The hypothesis is that Cilengitide masks, more effectively than GLPG0187, the RGD motif of ECM proteins to αV integrin, lowering latent TGF-β release and reducing VSMC phenotypic switch. Further structural and docking studies will be necessary to unveil the precise conformation and binding properties of αV integrin in the presence of Cilengitide and GLPG0187. This approach may also suggest the mechanism by which Cilengitide is more effective in preventing aortic dilation in both TAA models than GLPG0187. We hypothesize that the inefficacy of GLPG0187 relies on its inhibitory ability against all RGD-binding integrins. Lack of GLPG0187 inhibitory specificity may limit its potential, whereas the selectivity and specificity of Cilengitide may underlie its effectiveness.

Starting from the TAA context, the data in this paper may also exploit the potential of Cilengitide as a new pharmacological approach for fibrotic pathologies involving αVβ3 and αVβ5 integrins and still lacking an adequate therapeutic strategy. Indeed, drugs that nowadays reduce fibrosis in organs such as the lungs, kidneys, and heart have so far mainly focused on TGF-β inhibition. However, this approach is fraught with major side effects due to the pleiotropic physiological TGF-β effects and the evidence that TGF-β neutralization may even worsen disease phenotype (Cook et al., 2015). In contrast, tackling collateral mechanisms that contribute to the pathology may have significant anti-fibrotic efficacy, with limited side effects (Chen et al., 2016; Slack et al., 2022). Our results depict the inhibition of αV integrins as a promising strategy to limit collagen 1 production and thus fibrosis onset/progression. Several molecules have been designed to inhibit integrins by mimicking the RGD amino acid motif (Hatley et al., 2017), primarily in the oncology field. Cilengitide is one such molecule, having been tested in cancer clinical trials (Gerstner et al., 2015; Khasraw et al., 2016; Mason, 2015; Nabors et al., 2015; Stupp et al., 2014) and, therefore, extensively characterized in terms of pharmacokinetics, pharmacodynamics, efficacy, and safety in humans. Its repurposing for the treatment of TAA could therefore potentially be straightforward and translatable for clinical applications.

### Conclusions

In conclusion, this study further confirms the central role of αV integrins in TAA pathology and demonstrates a greater ability of Cilengitide in limiting TAA progression in comparison with a global αV integrin blockade strategy. The unique epidemiology of TAA makes Cilengitide a promising candidate for both a rare syndromic disease (such as MFS-TAA) and a common and widespread condition worldwide, such as non-syndromic sTAA. In this framework, Cilengitide may represent the first etiologic drug able to limit fibrosis in the treatment of TAA. Future studies will be necessary to determine for both types of TAA whether Cilengitide could be administered alone or in combination with other standard treatments for these patients (e.g., β-blockers, sartans, or angiotensin-converting enzyme inhibitors).

## Materials and methods

### Data availability

Data of this study are publicly available on Zenodo (https://doi.org/10.5281/zenodo.22707654). The spatial transcriptomics dataset is publicly available in the Gene Expression Omnibus (GEO) under accession no. GSE352523.

### Patient enrollment

During surgical replacement of TAAs with prosthetic grafts, surgeons collected both aneurysmal (dilated) tissue and a small portion of adjacent non-aneurysmal (non-dilated) tissue from the ascending thoracic aortas of patients with either sTAA or MFS.

Samples of sTAA patients were collected in the surgery room of Centro Cardiologico Monzino Istituto di Ricovero e Cura a Carattere Scientifico (IRCCS), while MFS patients’ specimens were collected in the surgery room of Azienda Socio Sanitaria Territoriale Fatebenefratelli-Sacco (ASST FBF-Sacco). All patients signed and gave written informed consent for tissue collection for research purposes, and the procedures were approved by the Ethics Committee of IRCCS Istituto Europeo di Oncologia e Centro Cardiologico Monzino (RE3001-CCM 1462) and ASST FBF-Sacco (Prot. No. 39138/2016). Sensitive data were handled in accordance with the Helsinki Declaration. Healthy ascending aortic samples were supplied by Tebubio and obtained from individuals who died of accidental causes (e.g., brain death), with no known cardiovascular risk factors or underlying cardiovascular diseases. Fresh biopsies were divided into fragments dedicated to histology, cell isolation, and molecular studies. Patients’ generalities have been reported in Table S1 A. Patients undergoing aortic repair, of either sex, aged 18 years or older, who have read and signed the informed consent have been included in the study. On the contrary, patients with previous aortic and/or mitral valve surgery, early menopause and/or osteoporosis, rheumatic heart disease, active malignancies, acute and chronic inflammatory states, chronic liver disease, chronic renal failure (creatinine >1.5 mg/dl), and thyroid-related diseases were not enrolled in the study.

### Spatial transcriptomics

Both dilated and non-dilated fragments of ascending aortas dedicated to histology analyses were fixed with Carnoy’s solution (60% ethanol, 30% chloroform, and 10% glacial acetic acid) and paraffin-embedded (CFPE). Firstly, RNA was extracted using the RNeasy FFPE Kit (Roche), following the manufacturer’s instructions. Then, the RNA integrity was evaluated with DV200 values on a TapeStation (Agilent) using the High Sensitivity RNA ScreenTape Kit (Agilent). Only CFPE vessel blocks with a DV200 >50 were included in the analysis. For spatial transcriptomics experiments, 3-μm-thick sections were cut from each aortic wall sample. The samples analyzed were *n* = 11 in total, including *n* = 2 HC aortas, *n* = 4 MFS aortas (*n* = 2 in the dilated region and *n* = 2 in the non-dilated area of the aneurysm), and *n* = 5 sTAA aortas (*n* = 2 in the dilated region and *n* = 3 in the non-dilated area of the aneurysm). After deparaffinization and rehydration, sections were stained with hematoxylin and eosin (Bio Optica); images were acquired using the AxioScan.Z1 (Carl Zeiss), and then the 10X Genomics protocol for the spatial transcriptomics assay was performed, according to the manufacturer’s guidelines. Libraries were constructed using the Visium Spatial for FFPE Gene Expression Kit (10X Genomics, cat. no. 1000336) and sequenced on a NextSeq2000 (Illumina) with a minimum sequencing depth of 50,000 read pairs per spatial spot. Sequencing was conducted as recommended (read 1: 28 cycles; i7 index read: 10 cycles; i5 index read: 10 cycles; read 2: 50 cycles), yielding 200–230 million reads per section. Reads were mapped to the GRCh38 reference genome and aligned using Space Ranger version 2.0.0 (10X Genomics). Downstream analysis was performed in R version 4.2.3 using Seurat version 5.1.0. Spots capturing fewer than 200 features were filtered, and the data were normalized using the SCTransform method. Clustering was performed with the Seurat functions FindNeighbors (top 30 dimensions) and FindClusters (resolution 0.3). Scores for pathways of interest were assigned to spots based on literature-derived gene sets using the AddModuleScore function. Spot composition inference was conducted by integrating single-cell RNA data from Tabula Sapiens Consortium et al. (2022) with the NNLS method in semla. Aggregated pseudobulk transcriptomic data were used to identify differentially expressed genes (DEGs) with EdgeR version 3.42.4, using the glmLRT test.

### Immunostaining and histological assays

5-μm-thick CFPE sections were rehydrated, incubated with primary antibodies against αV integrin (Abcam), COL1A1 (Cell Signaling Technology), anti-murine smad2/3 (Cell Signaling Technology), and anti-murine erk1/2 (Cell Signaling Technology) overnight (O/N) at 4°C, and secondary antibodies conjugated with Alexa Fluor fluorophores for 1 h at room temperature (RT). Nuclear staining was performed with DRAQ5 or Hoechst 33342 Buffer (Life Technologies). Slides were viewed with ApoTome2 equipped with an Axiocam camera (Carl Zeiss) and BC43 (Oxford Instruments) microscopes. Densitometric measurements were performed with ZEN Blue software (Carl Zeiss).

### Aortic VSMC isolation and treatment

Samples obtained from patients who underwent ascending thoracic aortic surgical substitution were abundantly washed with PBS for blood removal. Media and adventitia tunicae were physically separated, then tunica media was minced and digested O/N at 37°C in a solution of 2 mg/ml collagenase type II (Worthington Biochemical Corporation). The result of tissue digestion was filtered the day after with a 100-μm cell strainer, pelleted, and plated in Smooth Muscle Cell Growth Medium-2 SingleQuots (SmGM-2, Lonza). To improve cell growth, the day after isolation, the medium was changed to remove residual erythrocytes and ECM debris. For signalling and mechanotransduction experiments, VSMCs were treated with 5 ng/ml TGF-β1 (PeproTech) to mimic the pro-fibrotic environment, 0.5 µM Cilengitide (MedChemExpress), and 0.5 ng/ml GLPG0187 (Cayman Chemicals), a broad *pan*-αV integrin inhibitor.

### ImageStreamX imaging flow cytometry

For αV, β5, αVβ3, α5, and β1 integrin cellular localization experiments, VSMCs at low passages were acquired by the ImageStreamX imaging cytometer (Amnis Corporation), with 40× magnification and a low flow rate/high sensitivity using the INSPIRE software. Cells were gently detached from Petri dishes using TrypLE Select solution (Thermo Fisher Scientific) (Tsuji et al., 2017). Afterward, cells were pelleted, resuspended in FACS buffer (1× PBS, 0.1% BSA, and 5 mM EDTA), and incubated with conjugated primary antibodies for 10 min at RT. Data from 10,000 events per sample were collected, and the percentage of the positive elements was calculated using the IDEA software (Amnis Corporation). Primary antibodies against αV-FITC, β5-PE, αVβ3-APC, α5-APC, and β1-PE (Miltenyi Biotec) were used.

### Western blot

Both aortic tissues and cells were lysed in cell lysis buffer (Cell Signaling Technology) supplemented with protease and phosphatase inhibitor cocktails (Sigma-Aldrich). For signalling experiments, primary VSMCs at low passages were starved and treated with vehicle (complete medium), 5 ng/ml of TGF-β1 (PeproTech), 0.5 µM Cilengitide (MedChemExpress), and 0.5 ng/ml GLPG0187 (Cayman Chemicals). Similar treatments, without serum starvation, were administered to VSMC at low passages for the evaluation of mechanosensing mediators. Total protein extracts were subjected to SDS-PAGE and transferred onto a nitrocellulose membrane. The membranes were blocked for 1 h at RT in 5% nonfat dry milk in Wash Buffer (1× Tris buffer sulfate, 0.1% Tween 20) and then incubated O/N at 4°C with the appropriate primary antibody. Primary antibodies used were specific for: αV integrin (Abcam), COL1A1, phospho-SMAD2/3, SMAD2/3, phospho-ERK1/2, ERK1/2, RhoA, ROCK1, αSMA (Cell Signaling Technology), GAPDH (Santa Cruz Biotechnology), anti-murine phospho- and total smad2/3 (Cell Signaling Technology), anti-murine phospho- and total erk1/2 (Cell Signaling Technology), and p84 (Abcam). The membranes were incubated with peroxidase-conjugated secondary antibodies (GE Healthcare) for 1 h. Signals were visualized using the enhanced chemiluminescence western blotting detection system LiteUP and LiteAblot Extend (Bio-Rad Laboratories). Images were acquired with the ChemiDoc system, and densitometric analysis of membranes was performed using the Image Lab Software (Bio-Rad Laboratories). GAPDH was used as a protein loading control. To analyze functional RhoA levels, the Rho-GTPase Antibody Sampler Kit (Cell Signaling Technology, Danvers) was used following the manufacturer’s protocol.

### Immunofluorescence

Cells were seeded on round glasses, coated with recombinant human vitronectin protein (Gibco), and treated as previously described for 48 h. Then, the glasses were washed in PBS solution and fixed with 4% paraformaldehyde (Sigma-Aldrich) for 15 min. Cells were permeabilized with 1× PBS supplemented with 5% BSA and 0.1% Triton X-100 and then incubated O/N at 4°C with the primary antibody raised against αV integrin (Abcam) and COL1A1 (Cell Signaling Technology). The goat anti-mouse IgG (H+L) secondary antibody conjugated with AlexaFluor594 (Thermo Fisher Scientific) and goat anti-rabbit IgG (H+L) secondary antibody conjugated with AlexaFluor488 (Thermo Fisher Scientific) were incubated for 1 h at RT. Nuclei were stained with Hoechst 33342 Buffer (Life Technologies). Glasses were mounted on a slide with DAKO Mounting Medium for immunofluorescence (Sigma-Aldrich). Slides were viewed with an ApoTome2 microscope equipped with an Axiocam camera and analyzed with ZEN Blue software (Carl Zeiss).

### Animal models and aortic 2D echocardiography

Animal protocols were approved by the Italian Ministry of Health with the authorization no. 4/2022-PR. We adopted two different animal protocols, depending on the TAA model (i.e., syndromic or non-syndromic sTAA). 4-wk-old *Fbn1*^C1039G/+^ mice were used to model MFS-TAA (Habashi et al., 2006) and were purchased from The Jackson Laboratory, supplied by Charles River Laboratories Italia S.r.l. The sTAA model has been obtained by administering in drinking water the LOX inhibitor BAPN (BAPN, 1 g/kg/day) to 6-wk-old C57BL/6J WT mice for 21 days (Ren et al., 2016; Sawada et al., 2022). Both TAA models and littermate control (C57BL/6J) mice were treated three times a week, i.p., with either Cilengitide 90 mg/kg/day (MedChemExpress), GLPG0187 100 mg/kg/day (Cayman Chemicals), or vehicle (1% DMSO in PBS). The compound dosage has been selected on the basis of previous literature data (Nakamura et al., 2023; Roth et al., 2013). The i.p. injection has been preferred to allow a large volume of administration (200 μl) and a gradual systemic absorption. Both male and female animals were used. The number of animals was calculated using G*Power (version 3.1.9.2) software. Furthermore, a correction factor was added for each transgenic murine line to overcome the animal loss due to nonspecific mortality.

Animals were monitored with color-Doppler 2D echocardiography (Vevo3100, VisualSonics) equipped with a linear matrix transducer of 40 MHz (550D model). Transthoracic echocardiography was performed on mice sedated with 2% inhaled isoflurane (2-chloro-2-(difluoromethoxy)-1, 1, 1-trifluoroethane) (Baxter Healthcare Corporation) every 4 wk (MFS mice) or weekly (sTAA mice) to analyze aortic and cardiac function. Images of the aortic root and mid ascending aorta (halfway between the sinotubular junction and the brachiocephalic artery) were acquired in parasternal long-axis with an MS700 70 MHz Microscan transducer on the Vevo 3100 system (VisualSonics). The most dilated portion of the Valsalva’s sinus and ventriculo-aortic annulus were measured during end-diastole. The aortic measurement was performed by two blinded investigators in triplicate. Animals were euthanized by anesthesia with Ketamine 100 mg/kg at 16 wk of age (MFS mice) or at 9 wk of age (sTAA), and their organs were collected to perform morphometric, histologic, molecular, and biochemical analyses. For aortic root/ascending aorta samples, the aortic root was completely dissected from the left ventricle, cutting below the aortic valve and harvesting the region up to the brachiocephalic artery.

### mRNA extraction and qRT-PCR assay

Adventitial fibroblasts from HCs (HC-Fb) and sTAA patients (sTAA-Fb) were treated, as previously described for VSMC, with 5 ng/ml TGF-β1 (PeproTech), 0.5 µM Cilengitide (MedChemExpress), and 0.5 ng/ml GLPG0187 (Cayman Chemicals). Cellular RNA was isolated using a Total RNA Purification kit (Norgen Biotek Corp.). RNA quantification was determined with a spectrophotometer (ND-1000; NanoDrop, Euroclone). Reverse transcription was conducted with the SuperScript III (Invitrogen) following the manufacturer’s instructions. qRT-PCR was performed with the use of the iQTM SYBR Green Super Mix (Bio-Rad Laboratories). All reactions were performed in a 96-well format with the Bio-Rad C1000 Touch CFX96 system (Bio-Rad Laboratories). The relative quantities of specific mRNA were obtained with the use of the comparative Ct method and were normalized to the Ribosomal Protein L32 (RPL32).

### Masson’s trichrome staining

5-μm-thick sections of CFPE were cut from each sample. Sections were rehydrated, and Masson’s trichrome staining kit (Bio-Optica) was performed, following the manufacturer’s protocol. Slides were viewed with an Axio Imager microscope (Carl Zeiss). Densitometric analysis measurements were performed with ZEN Blue software (Carl Zeiss) by two blinded readers.

### Statistical analyses

Quantitative results are expressed as mean ± SD. Statistical significance was evaluated with GraphPad Prism 10. The comparisons between variables were analyzed by one-way ANOVA with Tukey’s post hoc test or two-way ANOVA with Bonferroni’s post hoc test, as appropriate. A value of P < 0.05 was deemed statistically significant.

### MTT assay

Cells were seeded in a 96-well multiwell plate. The day after, cells were washed with PBS, treated with integrin inhibitors, and incubated for 3 h in a humidified atmosphere (37°C, 5% CO_2_) with MTT solution (0.5 mg/ml of MTT in Opti-MEM medium, Thermo Fisher Scientific, Waltham). Cells were incubated for 10 min with solubilization solution (DMSO), and optical density was determined using a spectrophotometer plate reader at dual wavelengths of 590–620 nm (TECAN).

### Annexin V/ethidium bromide assay

Cells were seeded in a 96-well multiwell plate. The day after, cells were washed with PBS, treated with integrin inhibitors, and incubated with a solution of Annexin V-FITC (BD Biosciences) conjugated with fluorescein isothiocyanate (FITC) or ethidium bromide (EtBr) in the Incucyte Live-Cell Analysis System (Sartorius) for 6 days. Cells treated with 980 mM H_2_O_2_ in PBS were used as positive control samples. Four pictures/well were taken every 2 h.

### ELISA assay

Protein concentration was determined by a bicinchoninic acid assay. Human TGF-β1 ELISA kit (R&D Systems) was used following the manufacturer’s protocol. Cells were treated with 0.5 µM Cilengitide (MedChemExpress) or with 0.5 ng/ml GLPG0187 (Cayman Chemicals) for 48 h. Supernatant of cells was used for the assay (in triplicate). Results were collected with a spectrophotometer plate reader at a wavelength of 450 nm (TECAN).

### ITGAV gene knockdown in VSMC

To downregulate ITGAV gene expression, a pGFP-C-shLenti vector containing an expression cassette (short hairpin RNA), a puromycin resistance gene, and a tGFP gene between the 5′LTR and 3′LTR sequences was used. Cells were seeded in a multiwell plate, infected with four different lentiviral particles containing 4 distinct human shRNA (TL312085VA 5′-CTA​CTT​GAC​TGT​GGT​GAA​GAC​AAT​GTC​TG-3′; TL312085VB 5′-GAG​CTG​AGA​AAC​AAT​GGT​CAA​GTT​CAT​T-3′; TL312085VC 5′-TCA​GTG​GTT​TGG​AGC​ATC​TGT​GAG​GTC​GA-3′; TL312085VD 5′-GCA​CCA​GCA​GTC​AGA​GAT​GGA​TAC​TTC​TG-3′, AMS Biotechnology), and treated for 48 h. Negative controls were also generated by using a lentiviral particle with a control scramble shRNA (TR30021V). Puromycin (Sigma-Aldrich) was then added to positively select only the infected cells. At the end of the infection protocol, cells were opportunely treated and then lysed for western blot assays.

### Online supplemental material

Table S1 provides the clinical characteristics of the study population and DEGs related to ECM and mechanotransduction. Fig. S1 shows spatial transcriptomic analyses of integrin expression and integrin gene feature plots in VSMC. Fig. S2 reports spatial expression of ECM genes and the protein–protein interaction network connecting *FBN1* to genes deregulated in MFS-VSMC. Fig. S3 provides VSMC characterization and complementary in vitro characterization of Cilengitide and GLPG0187 features in terms of cytotoxicity, apoptosis/necrosis, and TGF-β1 release. Fig. S4 shows spatial expression of mechanotransduction-related genes and characterization of *ITGAV* knockdown in MFS-VSMC, including its effects on TGF-β-related signalling.

## Ethics statement

Informed consent was obtained from all subjects involved in the study. Written informed consent has been obtained from the patient(s) to publish this paper.

## Supplementary Material

Table S1 shows (A) features of MFS and sTAA patients involved in the study and (B) DEGs related to ECM proteins and mechanotransduction obtained by spatial transcriptomics analysis data.

SourceData F4 is the source file for Fig. 4.

SourceData F5 is the source file for Fig. 5.

SourceData F7 is the source file for Fig. 7.

SourceData FS4 is the source file for Fig. S4.

## Data Availability

The raw data of this manuscript are available in Zenodo (doi: 10.5281/zenodo.22707654) and GEO (accession no. GSE352523).
