# Supplementary material for: Cilengitide limits syndromic and sporadic thoracic aortic aneurysm by targeting specific αV integrin
Source: J Exp Med. 2026 Nov 11;223(12):e20260700. doi: 10.1084/jem.20260700 (PMC13753961; doi:10.1084/jem.20260700)

SourceData**F4** - Panel A (SMAD2/3)

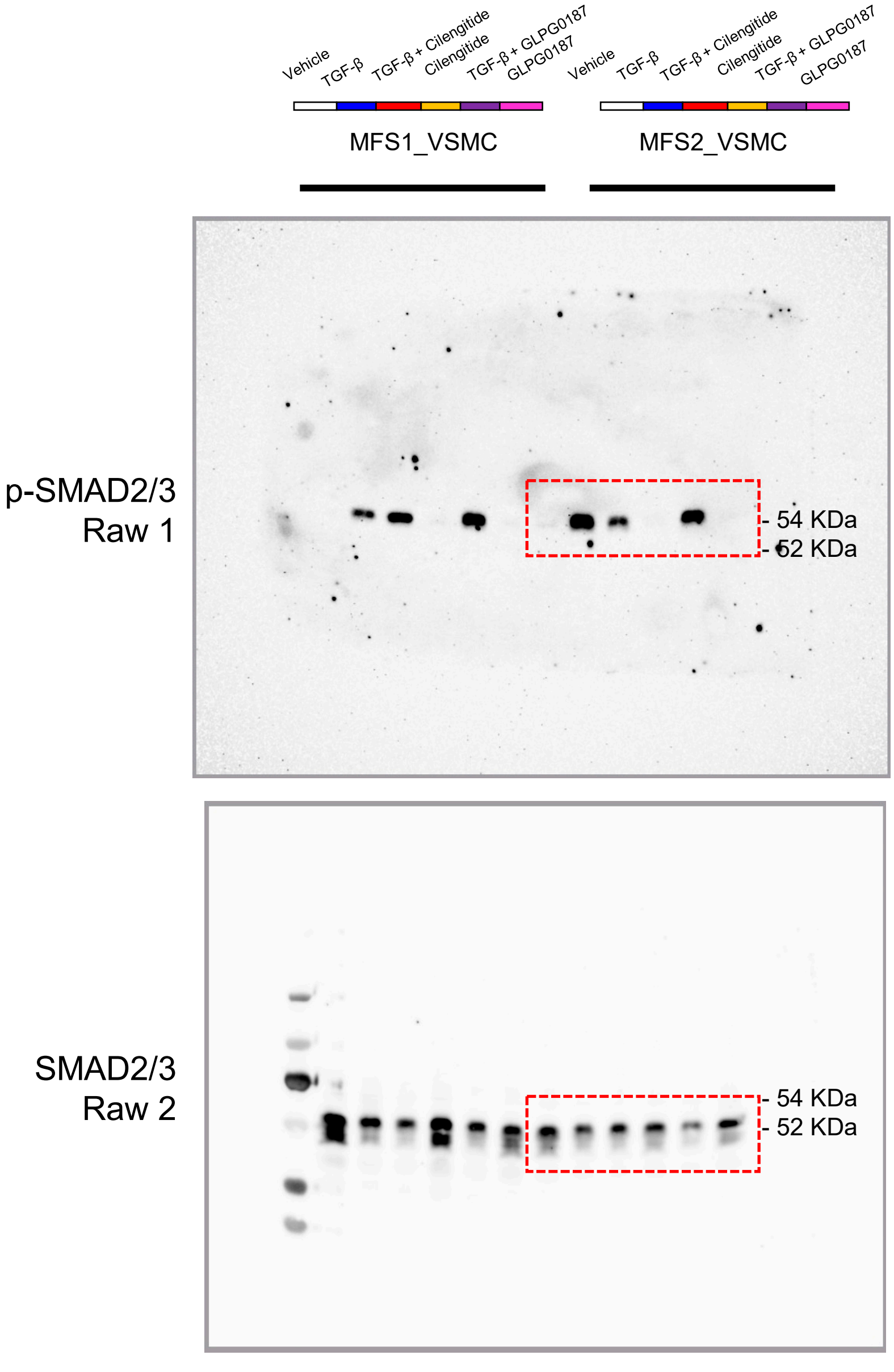

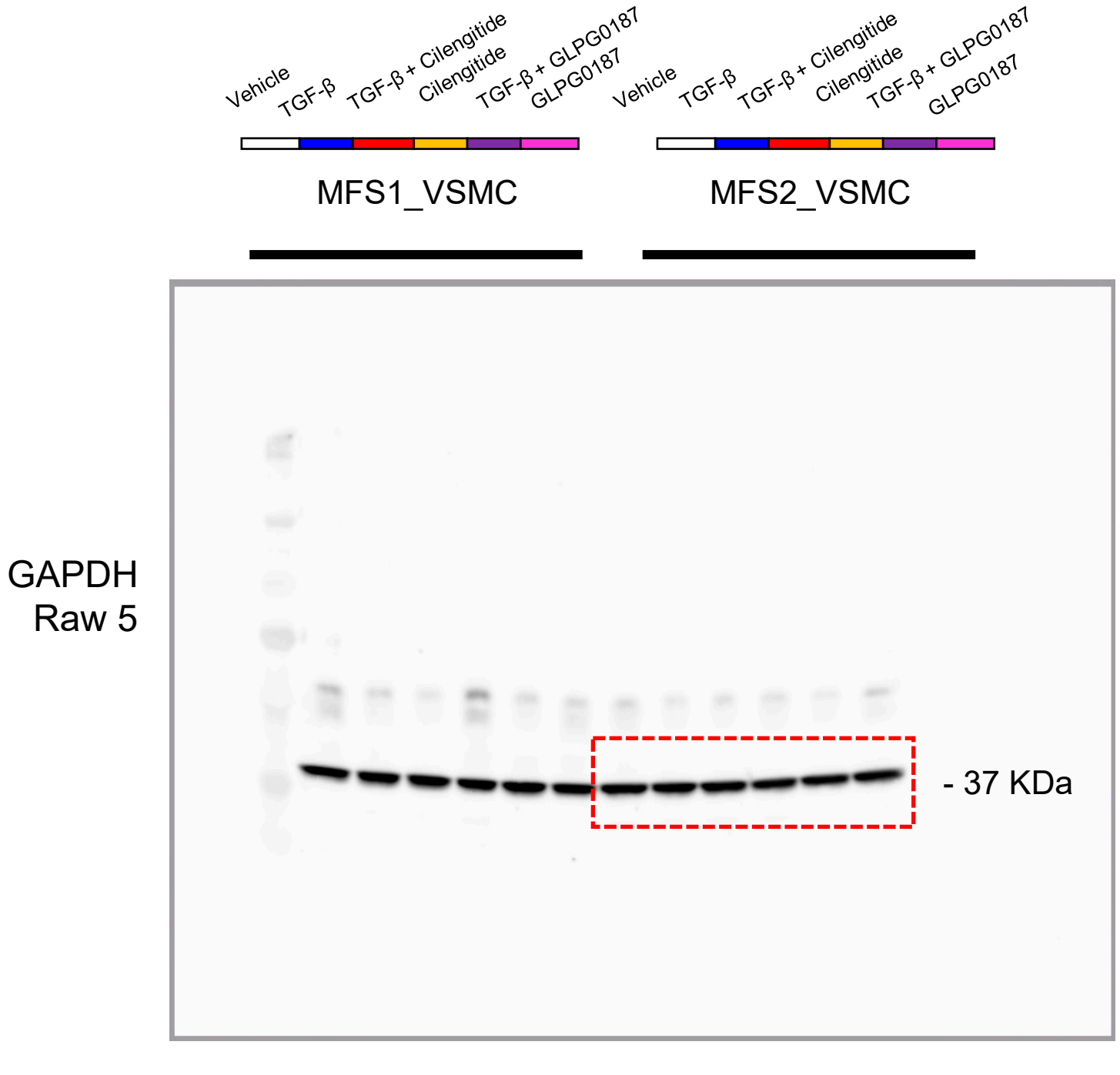

SourceData**F4** - Panel A (ERK1/2)

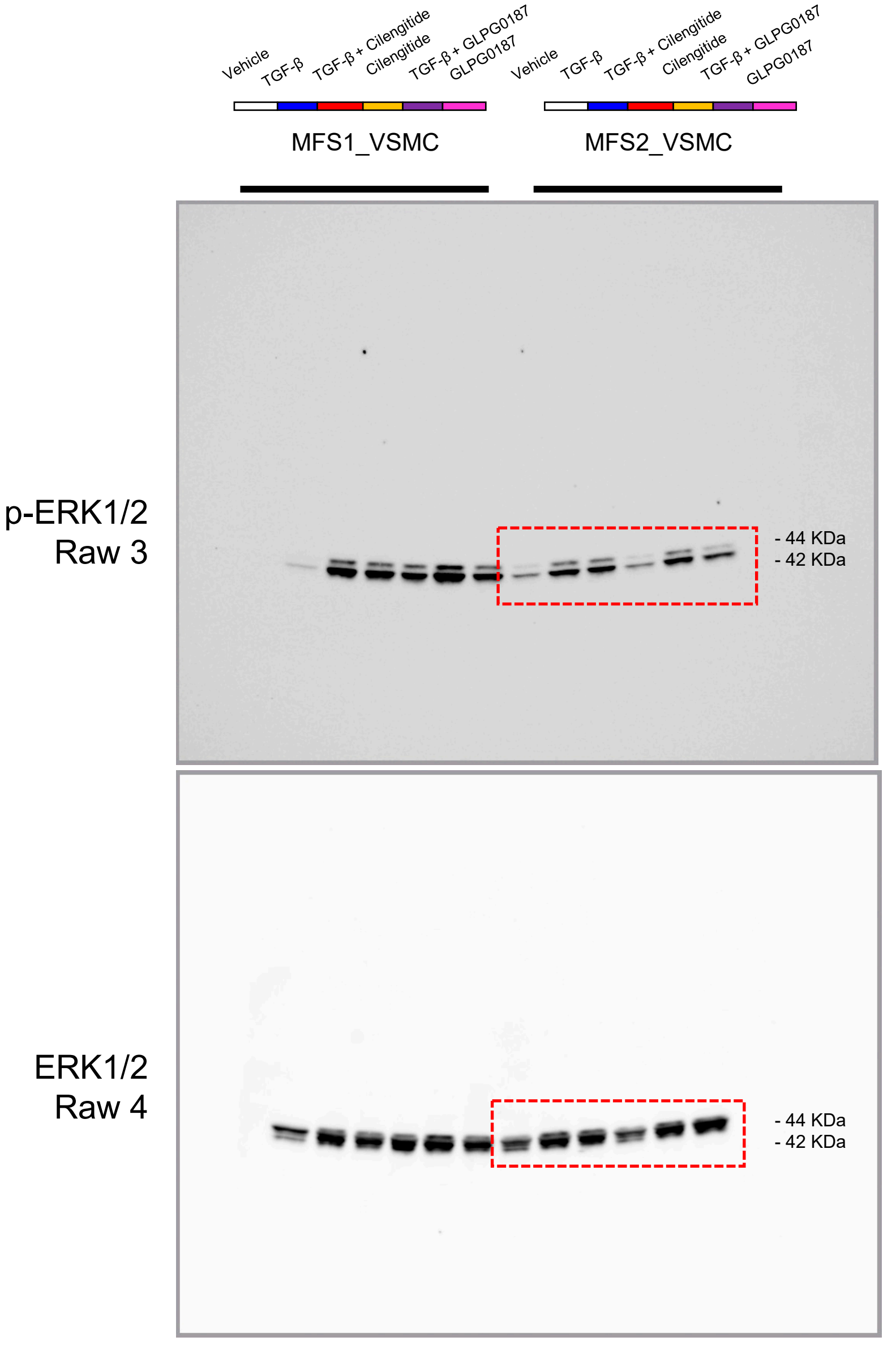

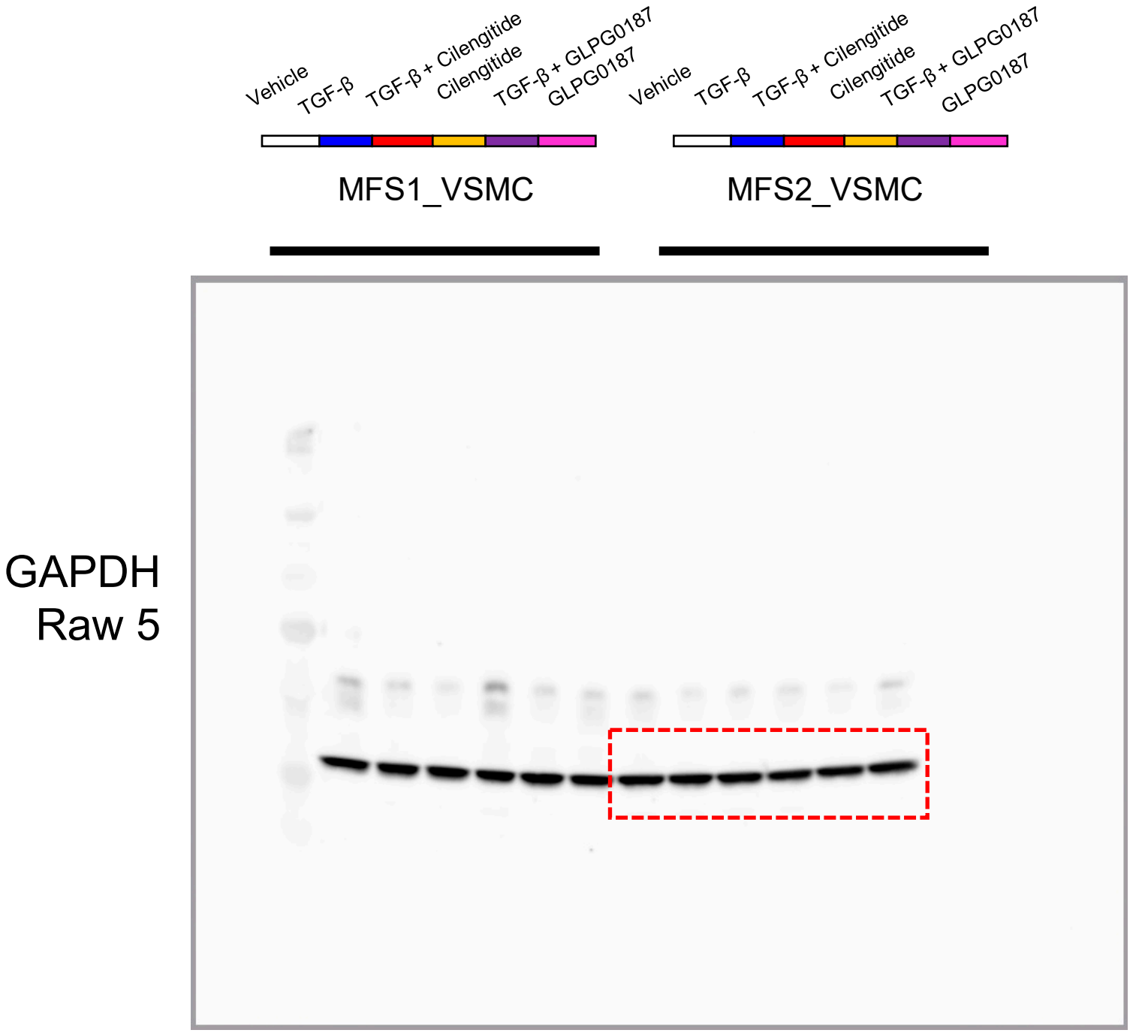

SourceData**F4** - Panel A (Rho GTPase, ROCK1, αSMA)

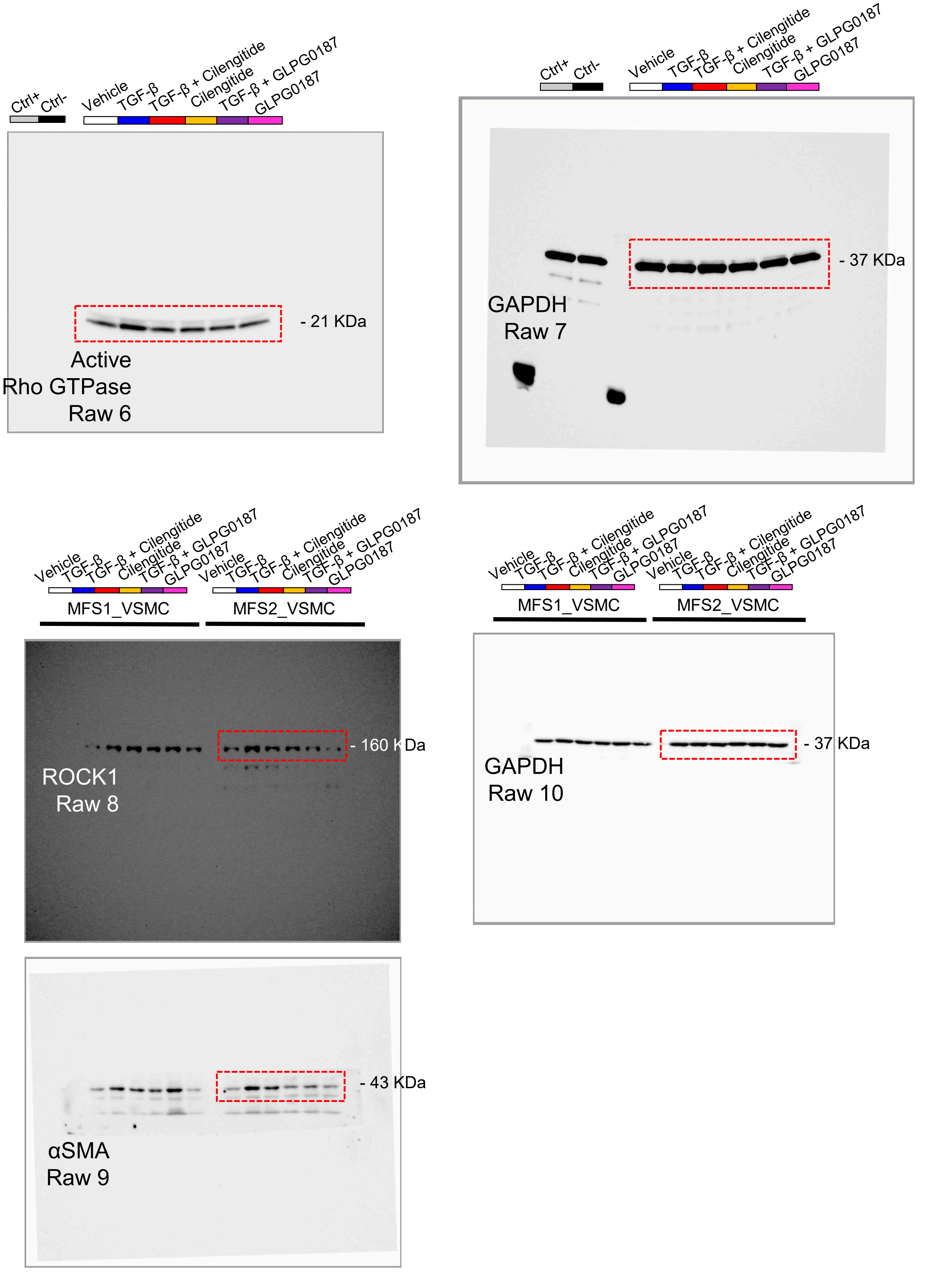

SourceData**F4** - Panel G (SMAD2/3)

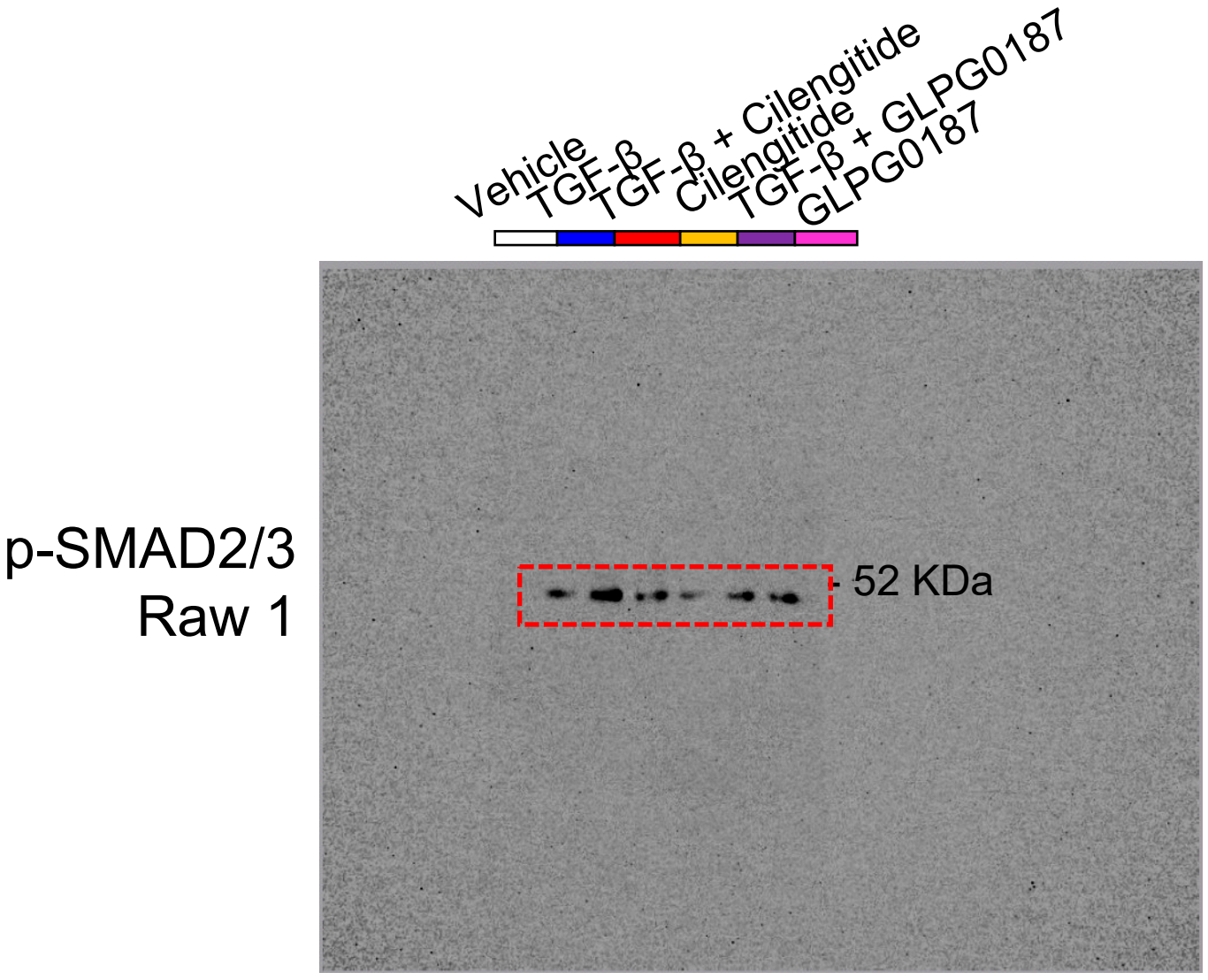

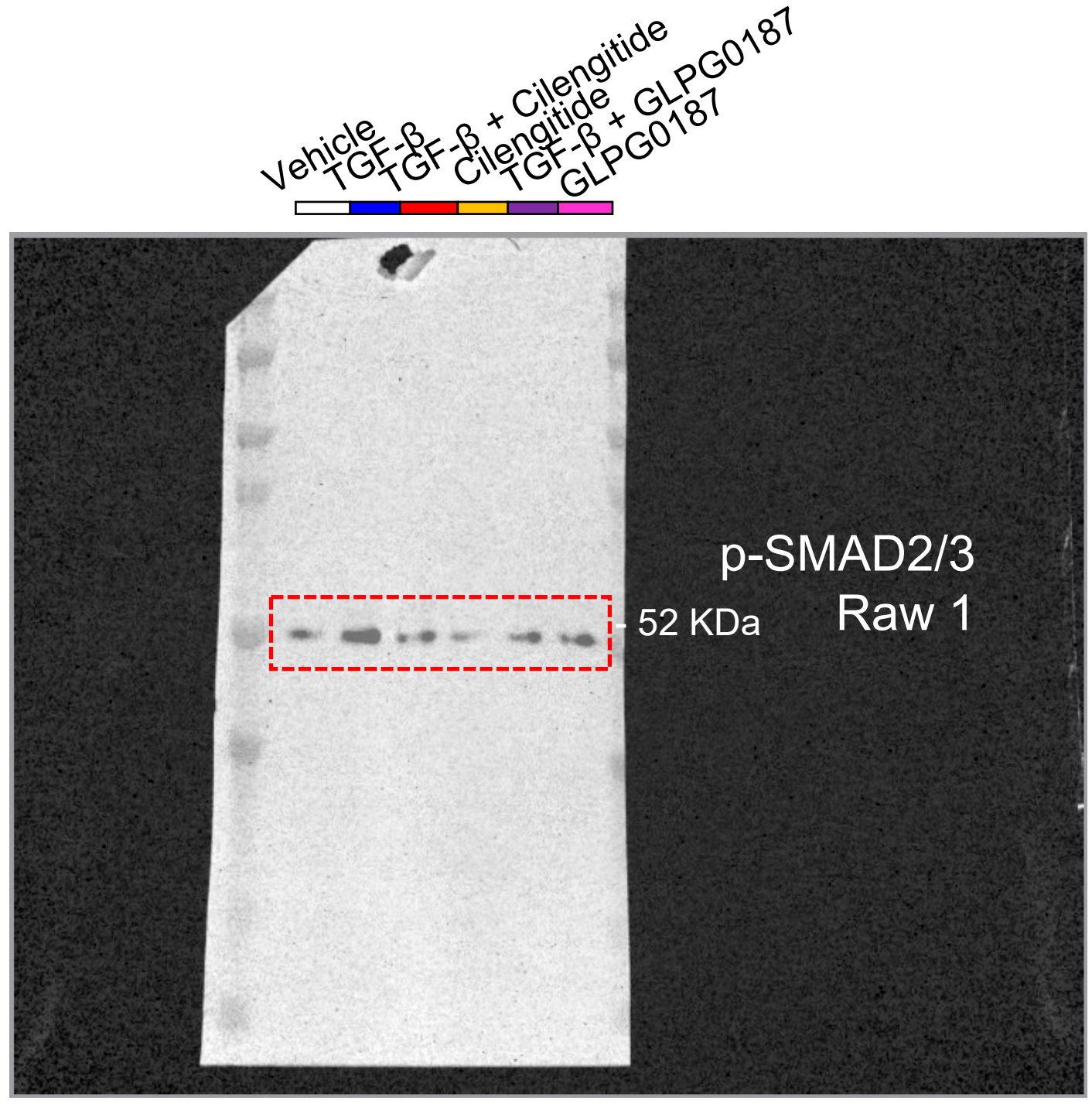

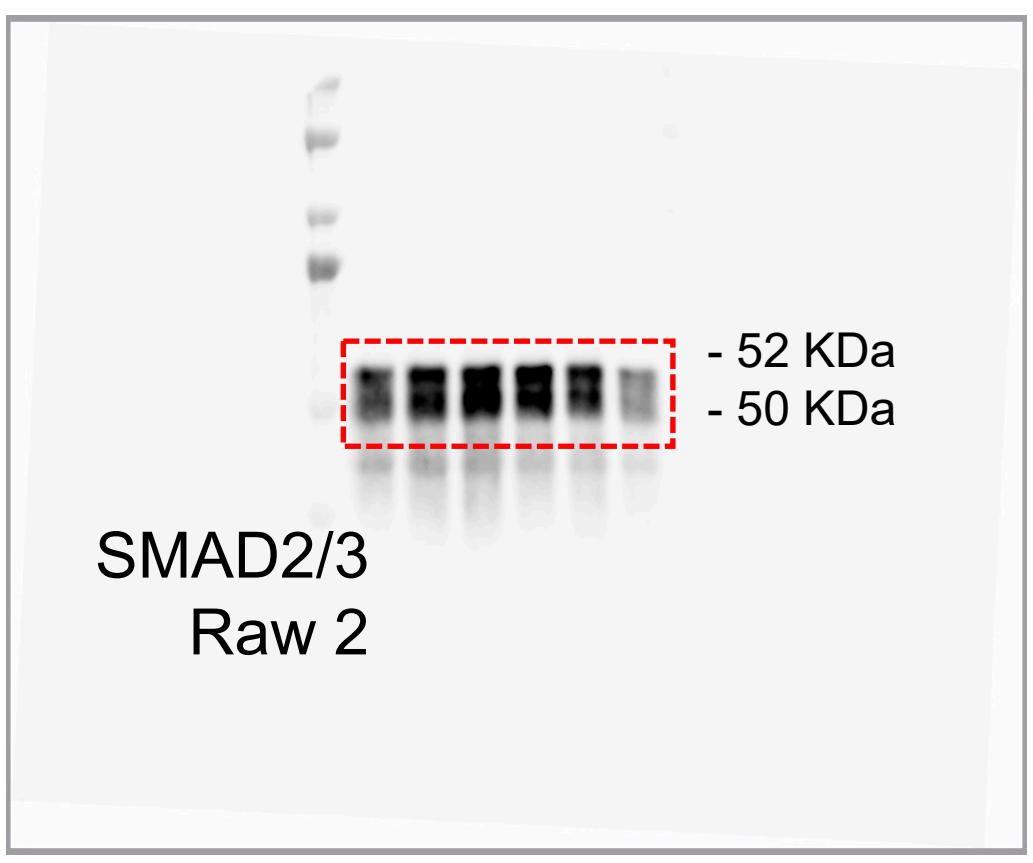

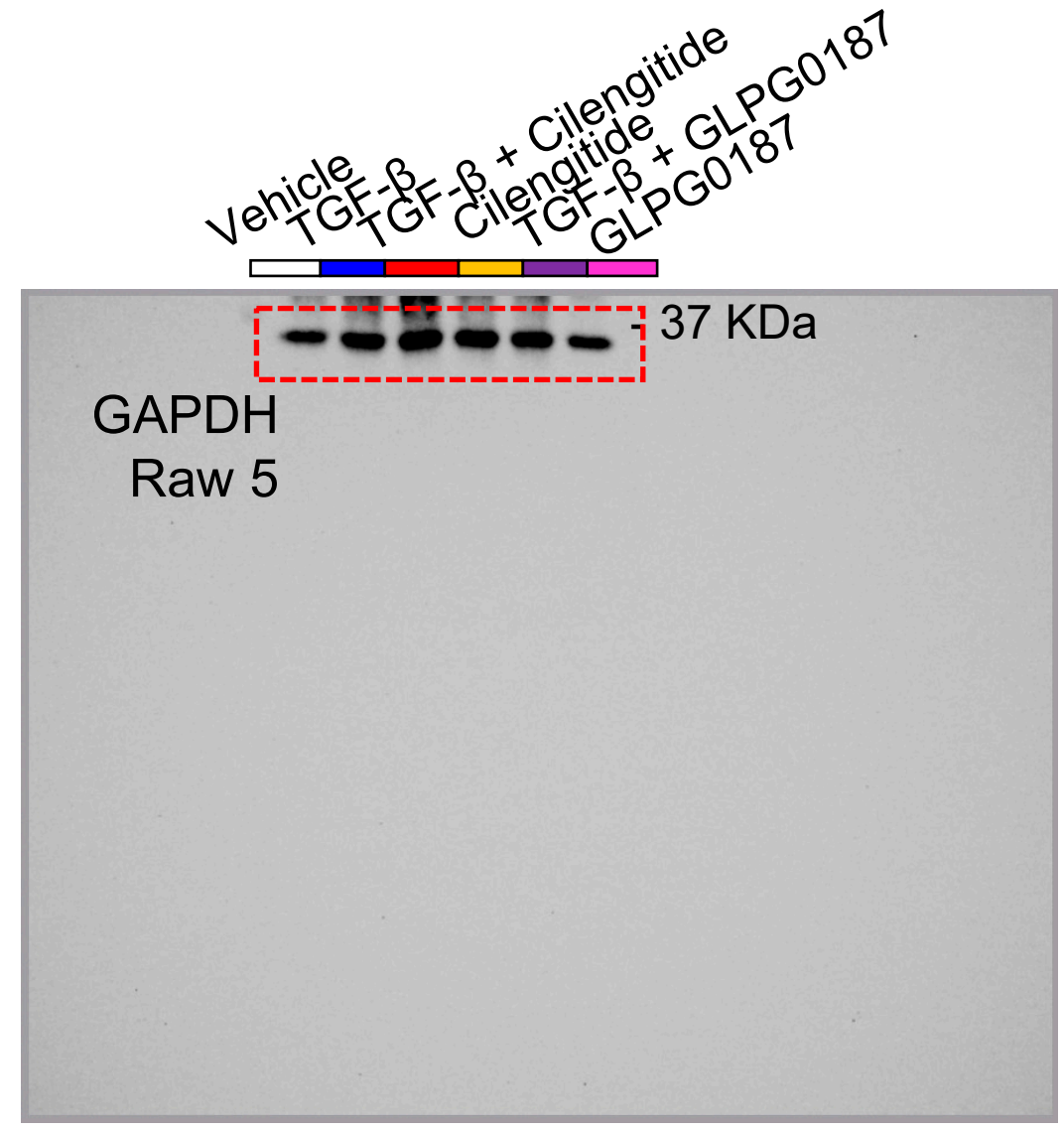

SourceData**F4** - Panel G (ERK1/2)

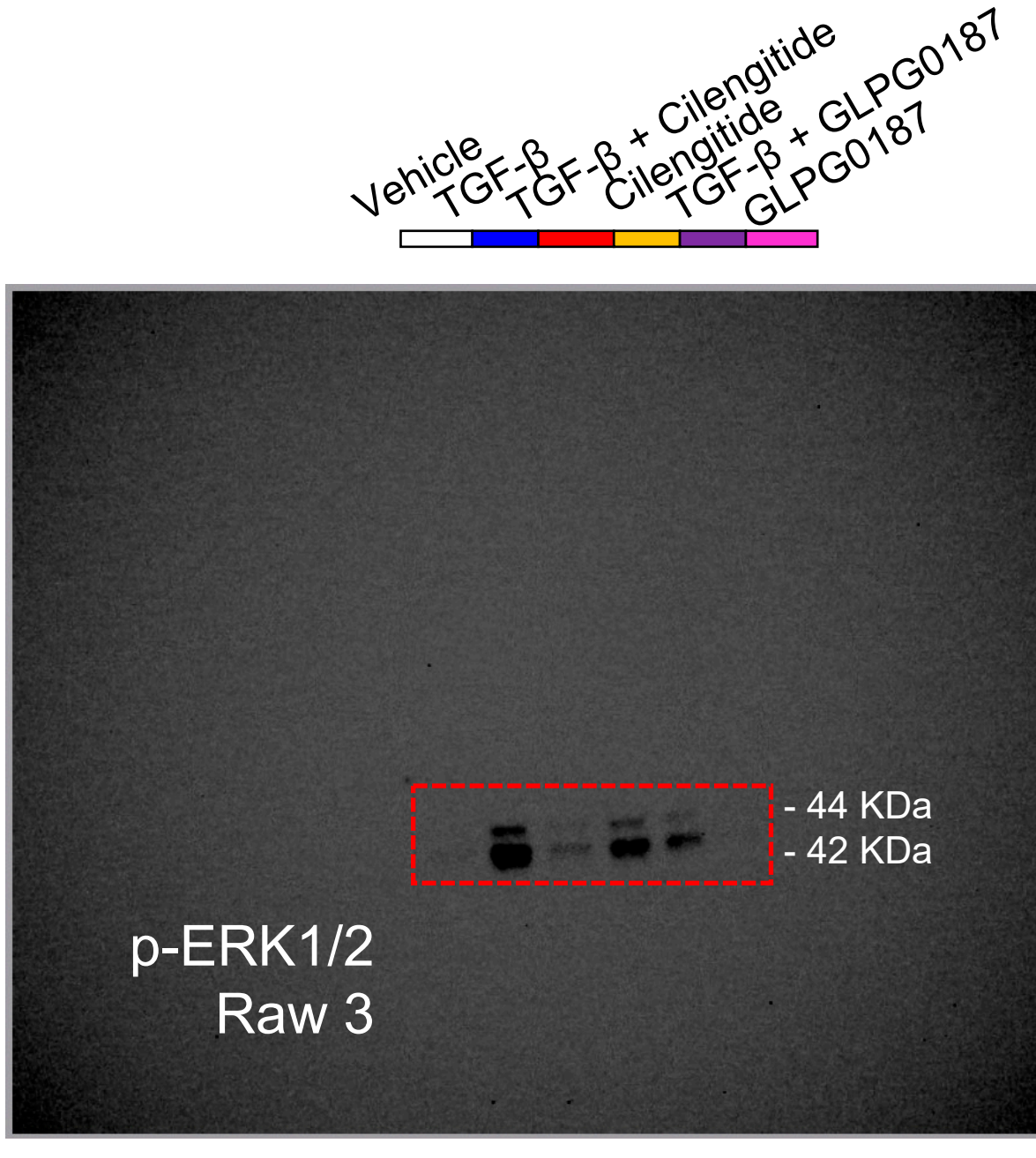

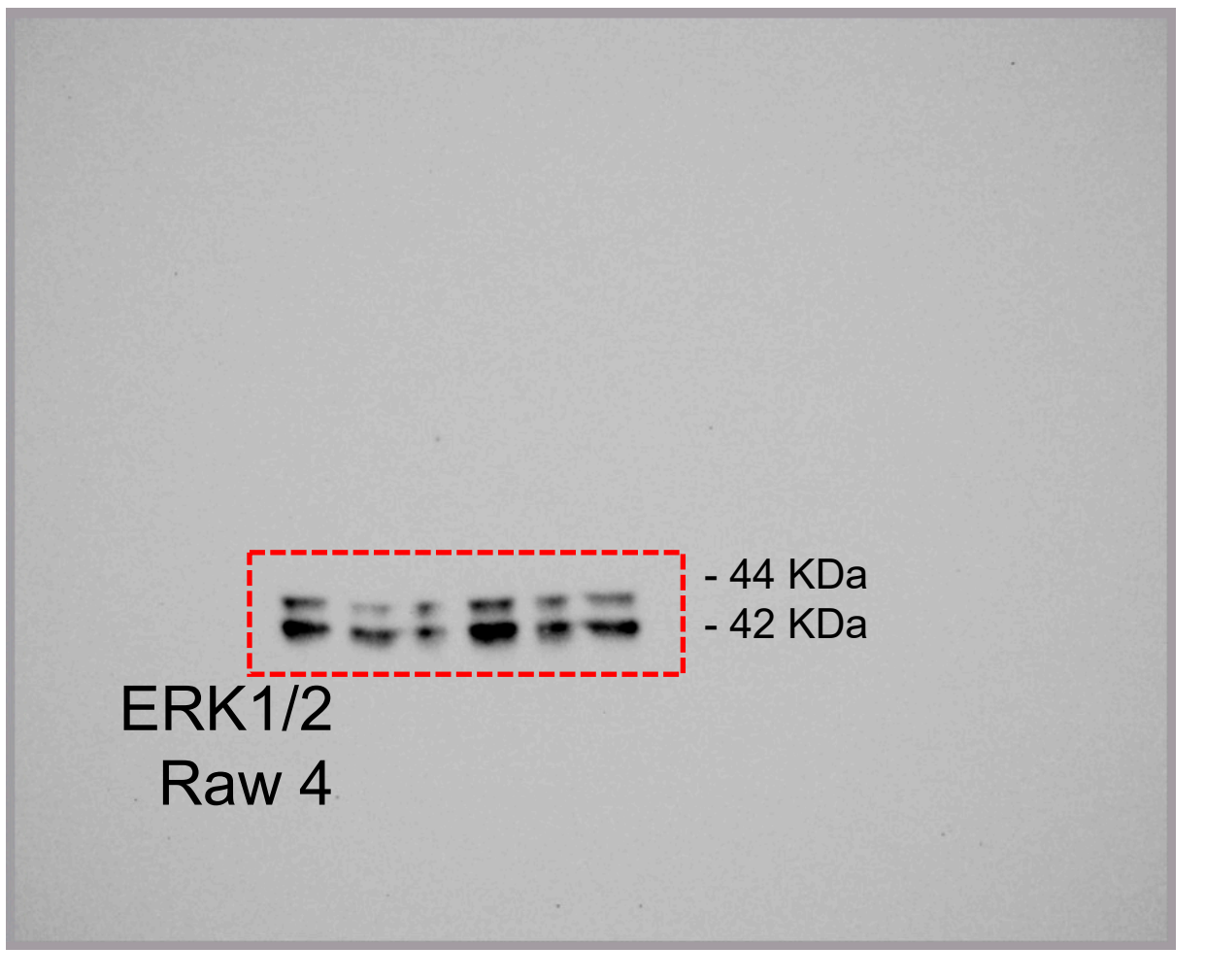

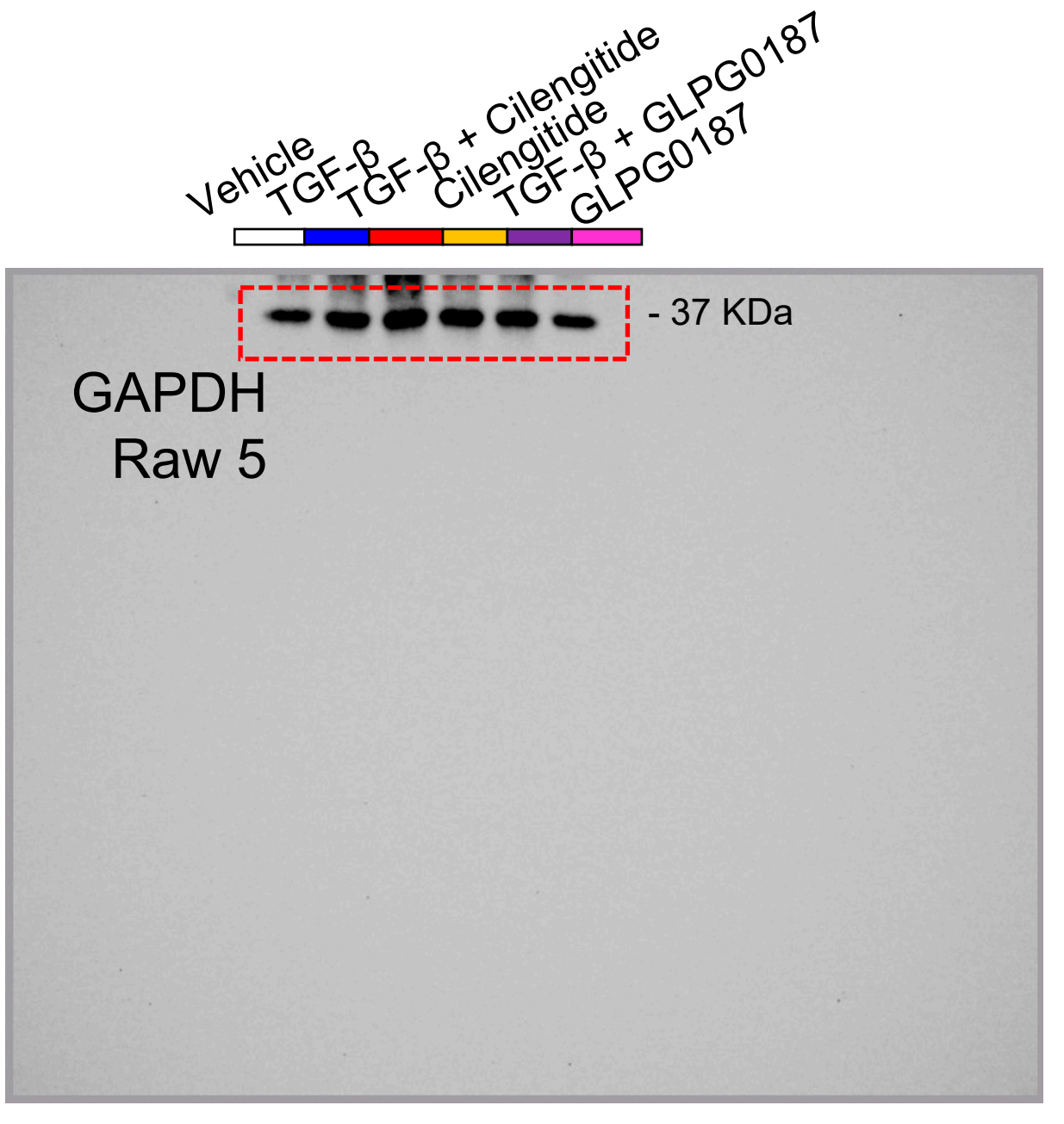

SourceData**F4** - Panel G (Rho GTPase, ROCK1, αSMA)

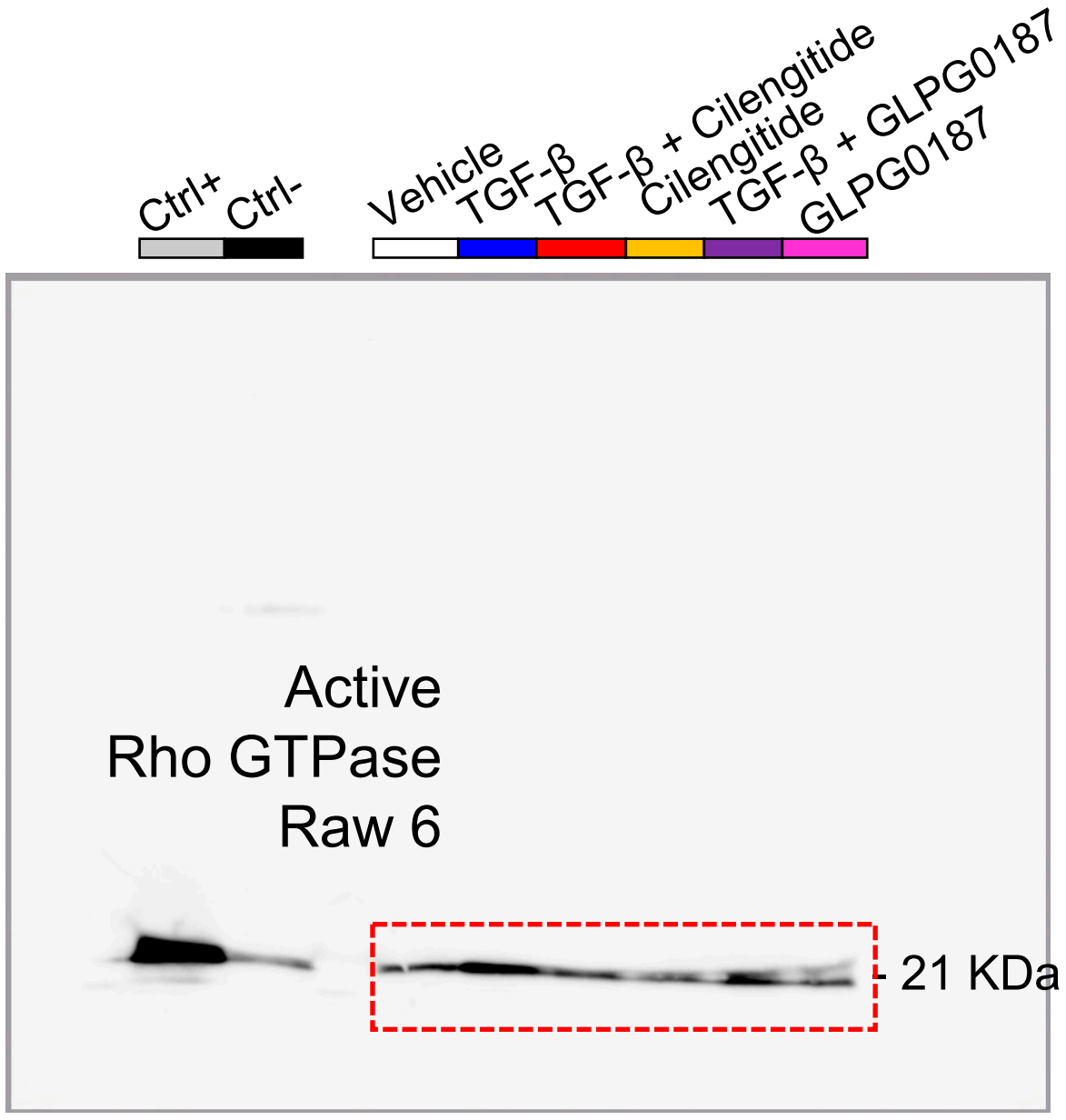

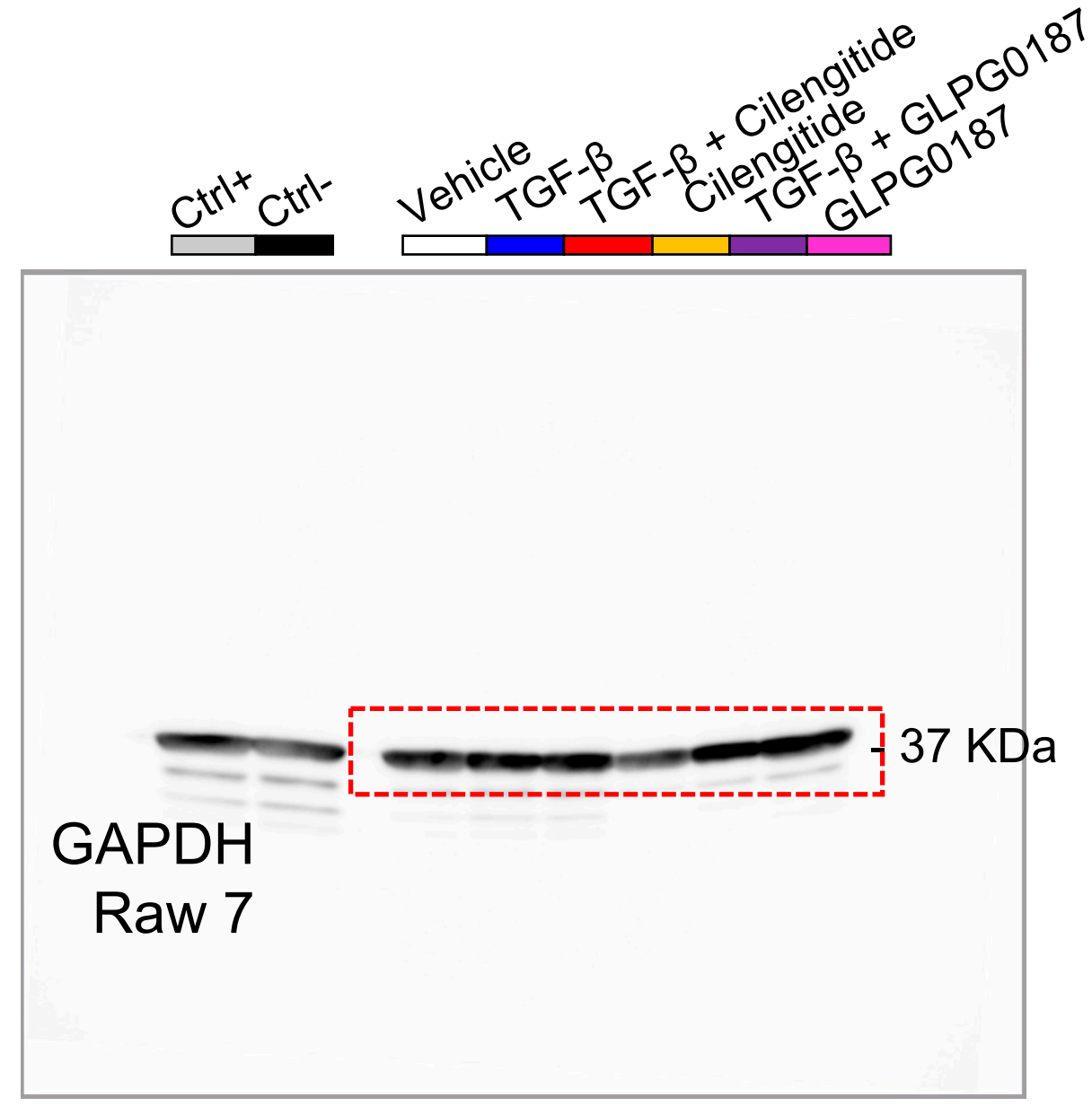

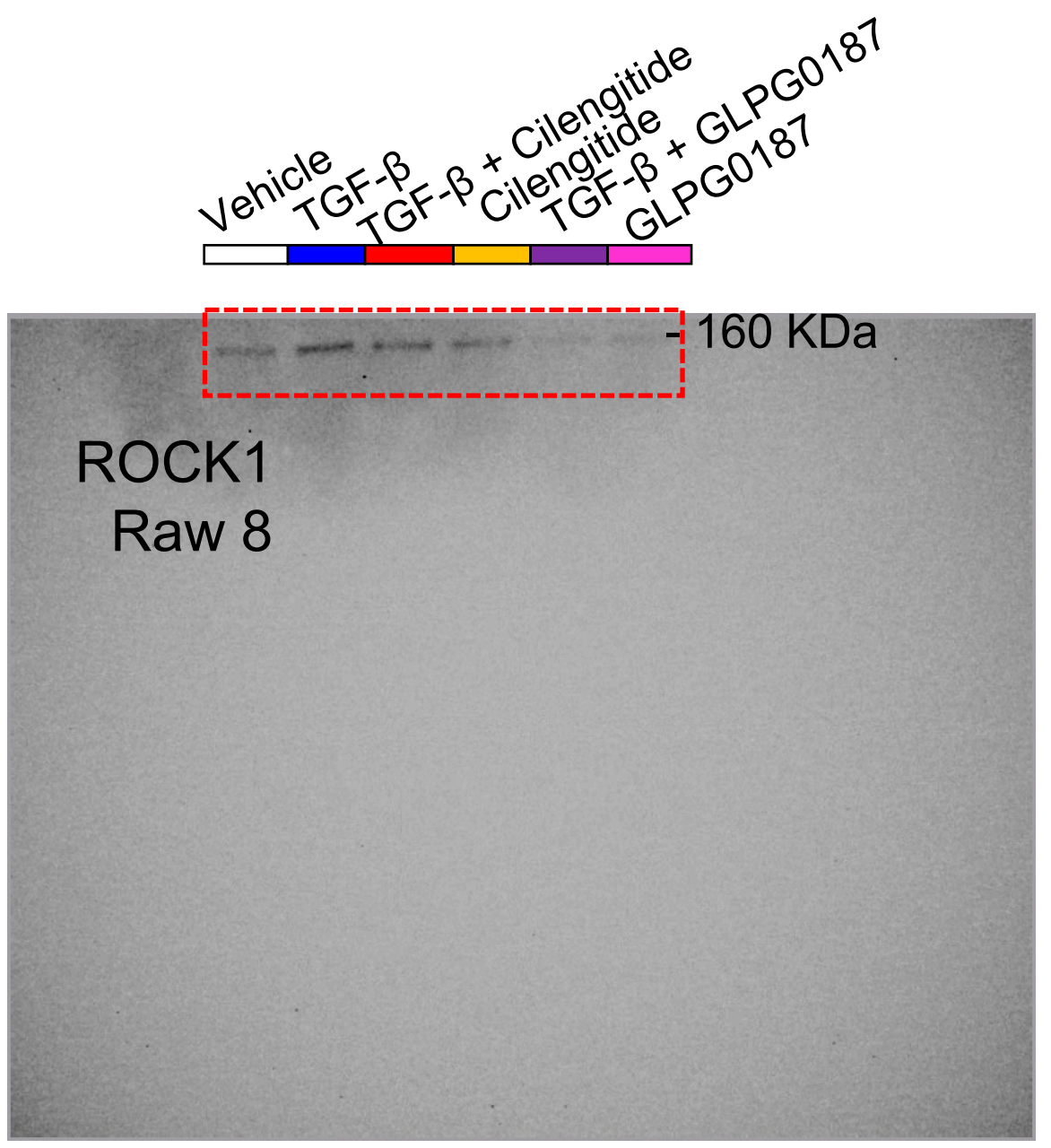

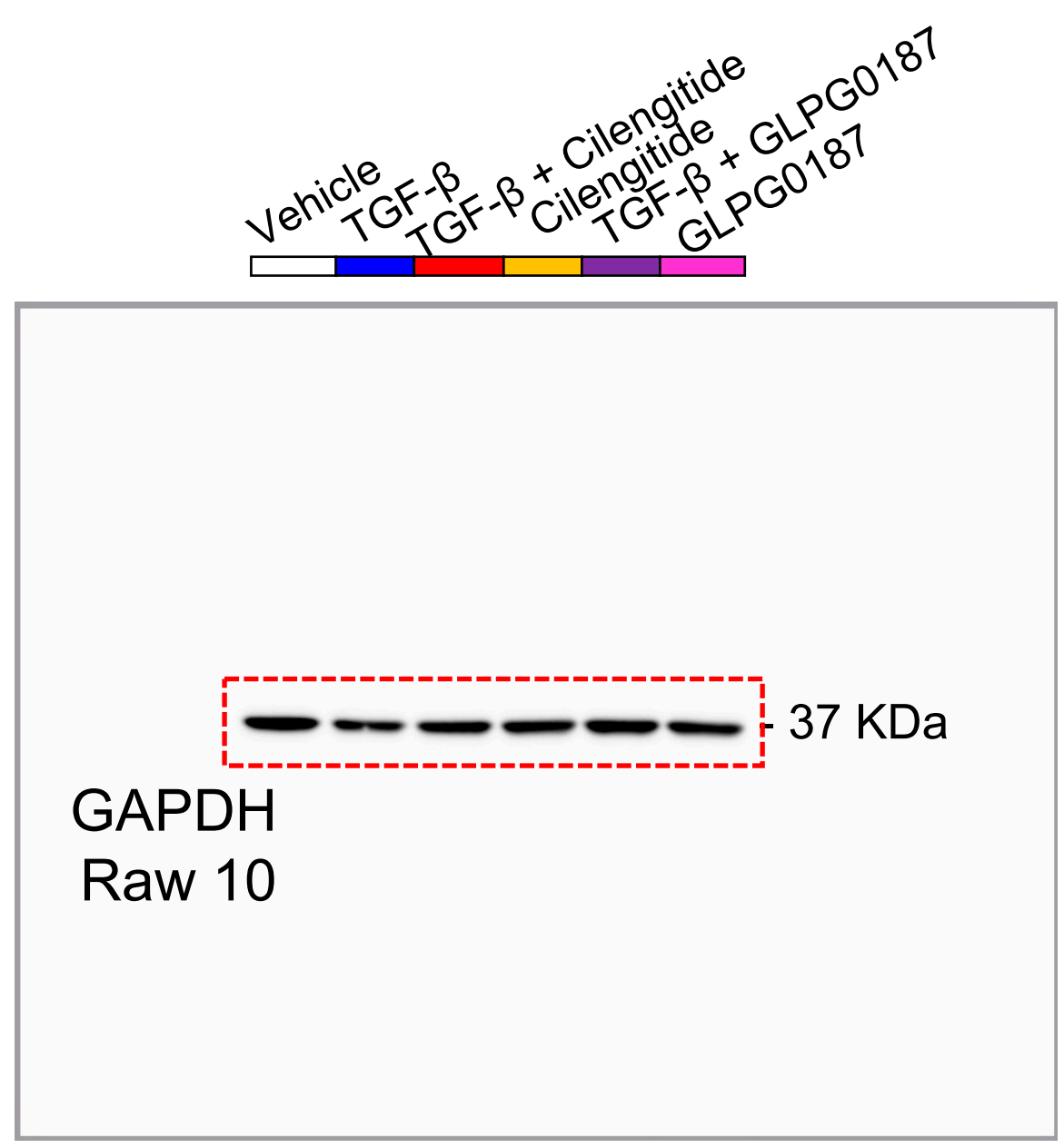

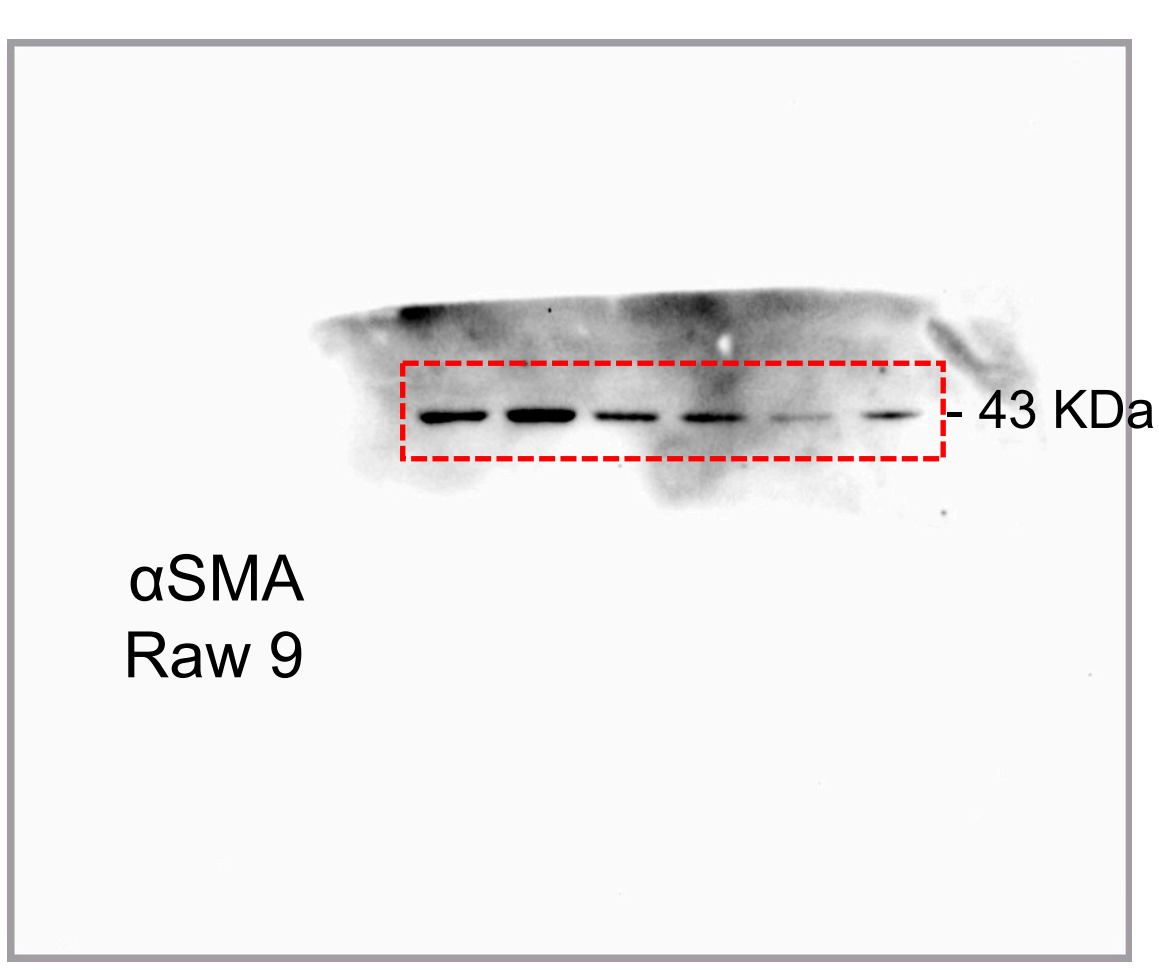

Supplement: Supplementary file 1 [file jem_20260700_sourcedataf4.pdf]
