# Supplementary material for: Cilengitide limits syndromic and sporadic thoracic aortic aneurysm by targeting specific αV integrin
Source: J Exp Med. 2026 Nov 11;223(12):e20260700. doi: 10.1084/jem.20260700 (PMC13753961; doi:10.1084/jem.20260700)

SourceData**F5** – Panel A (left side)

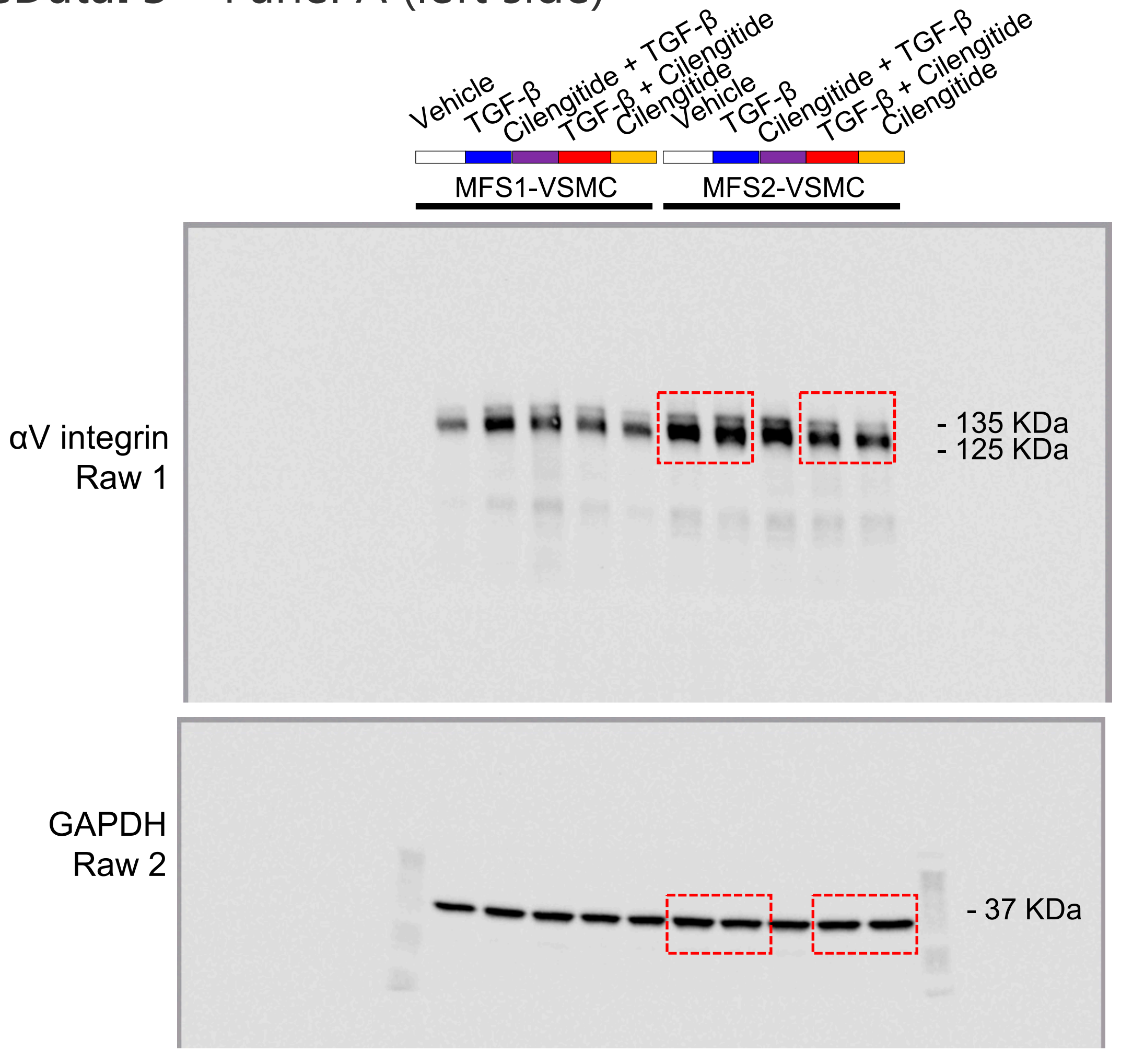

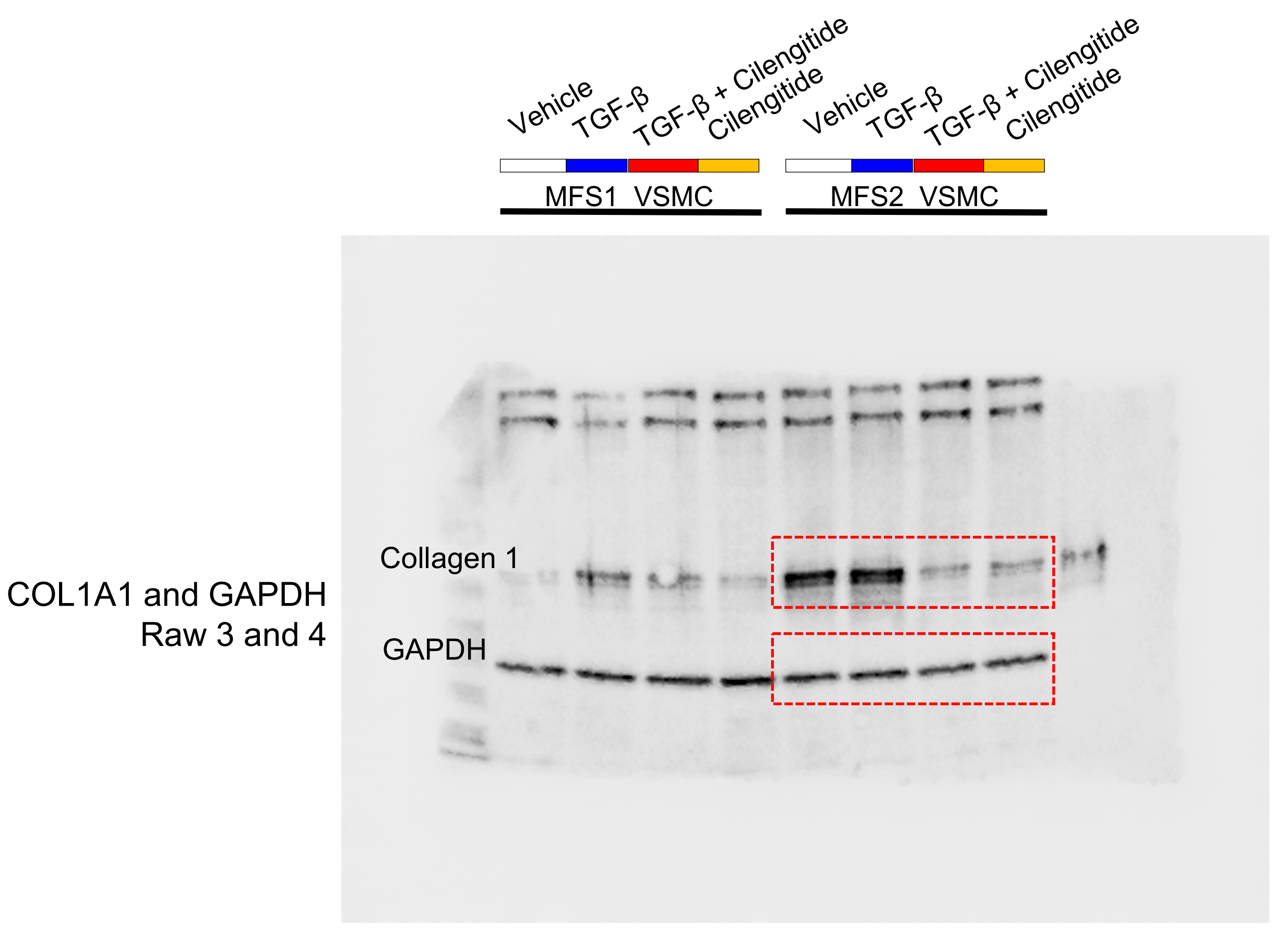

SourceDataF5 – Panel A (right side)

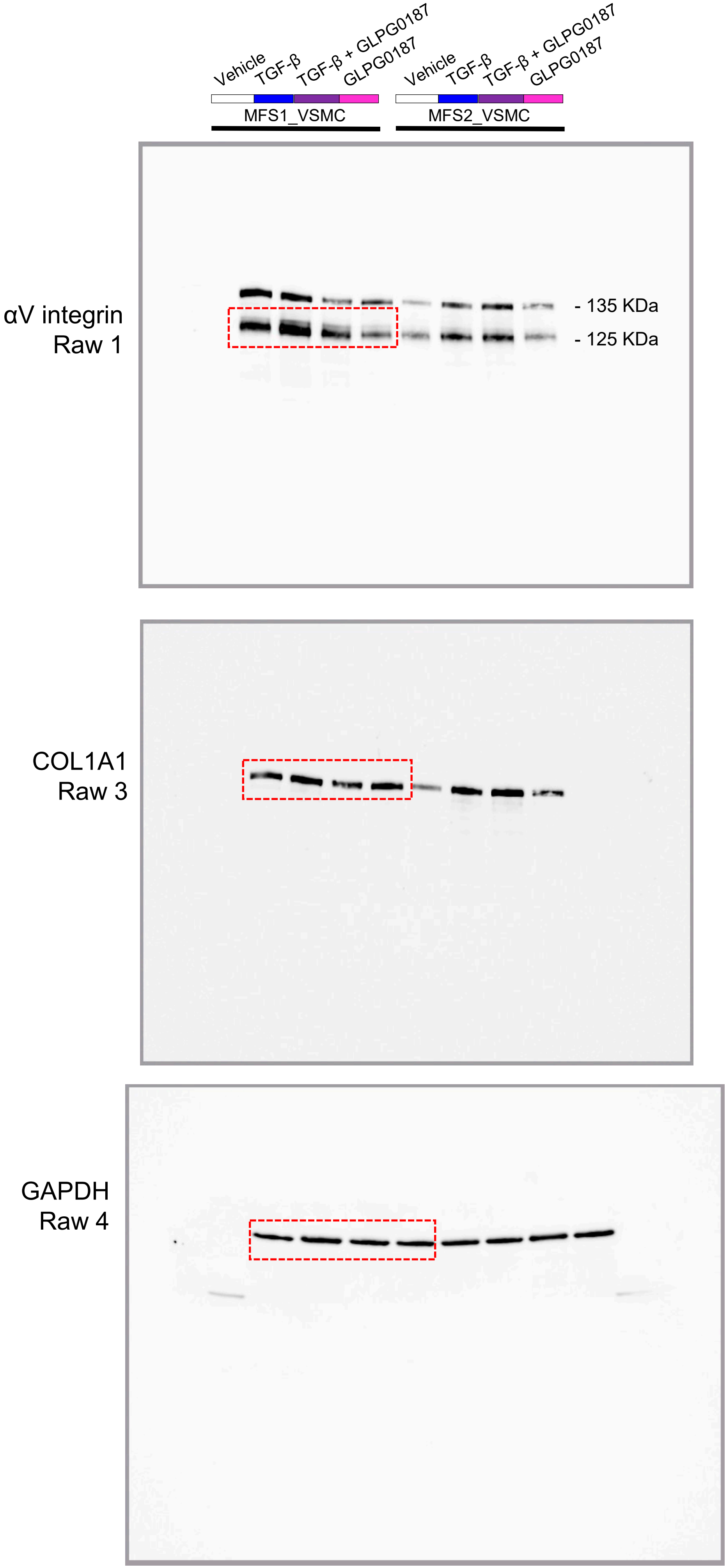

SourceData**F5** – Panel D

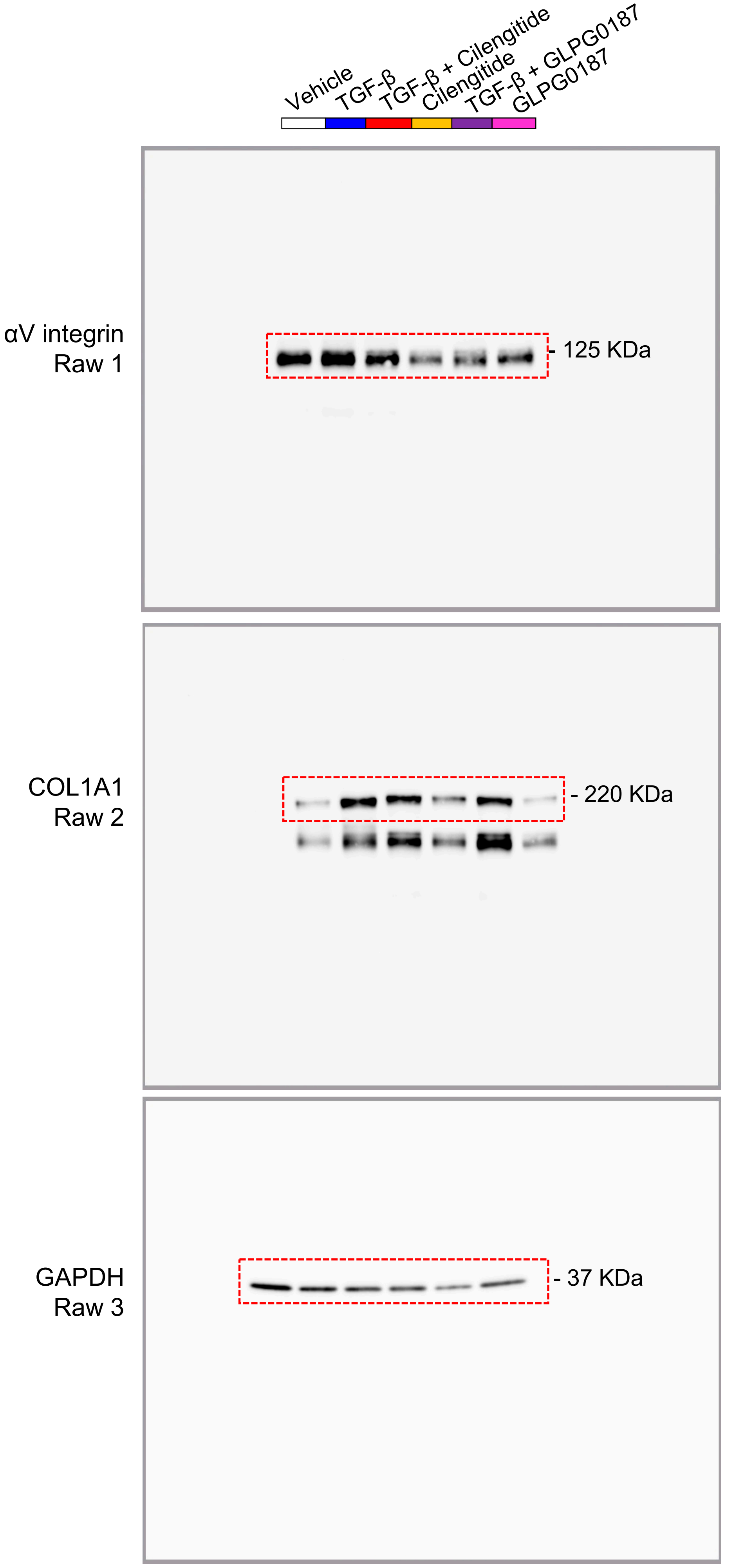

SourceData**F5** – Panel I

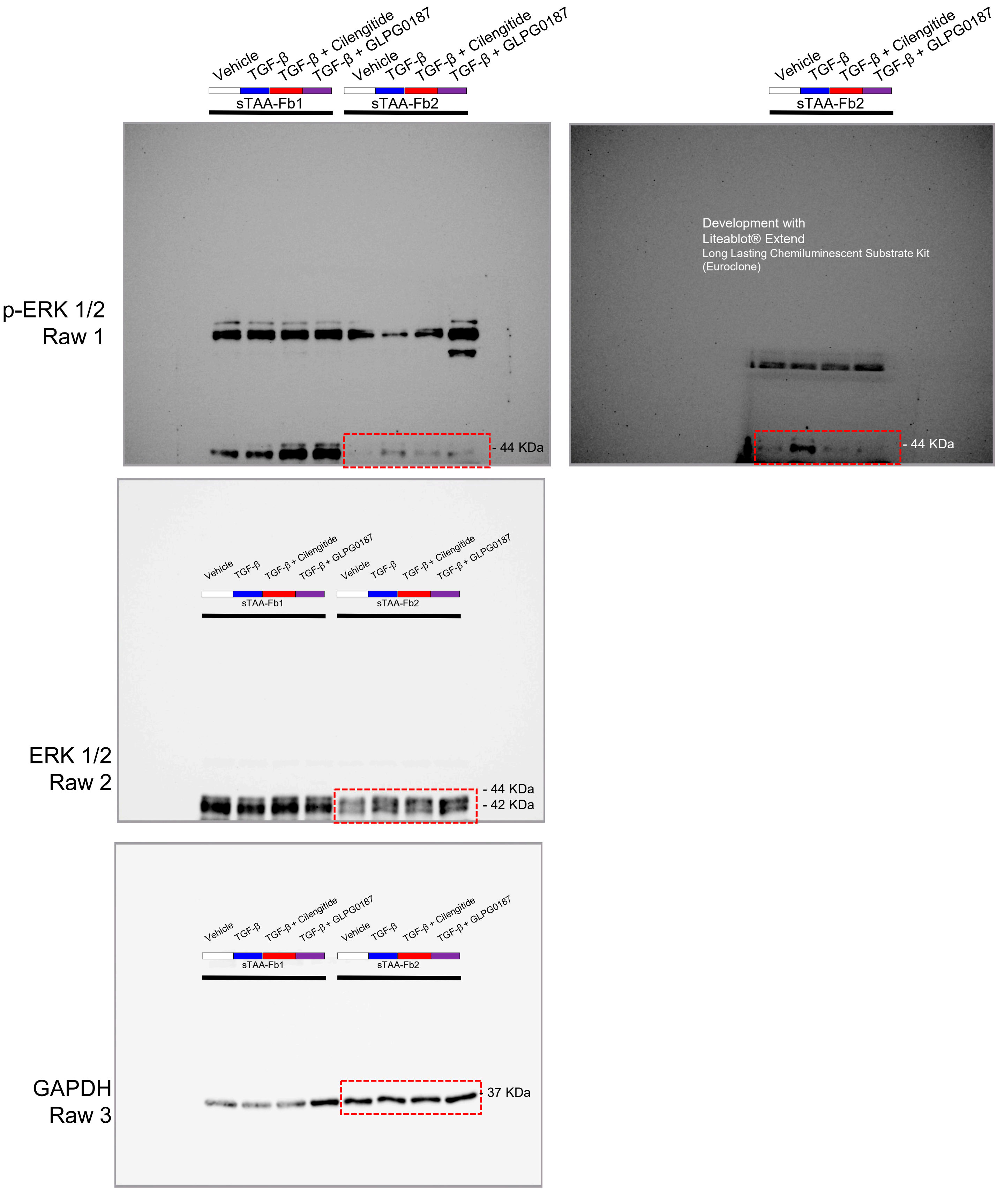

Supplement: Supplementary file 2 [file jem_20260700_sourcedataf5.pdf]
