# Supplementary material for: Cilengitide limits syndromic and sporadic thoracic aortic aneurysm by targeting specific αV integrin
Source: J Exp Med. 2026 Nov 11;223(12):e20260700. doi: 10.1084/jem.20260700 (PMC13753961; doi:10.1084/jem.20260700)

SourceData**FS4** – Panel E

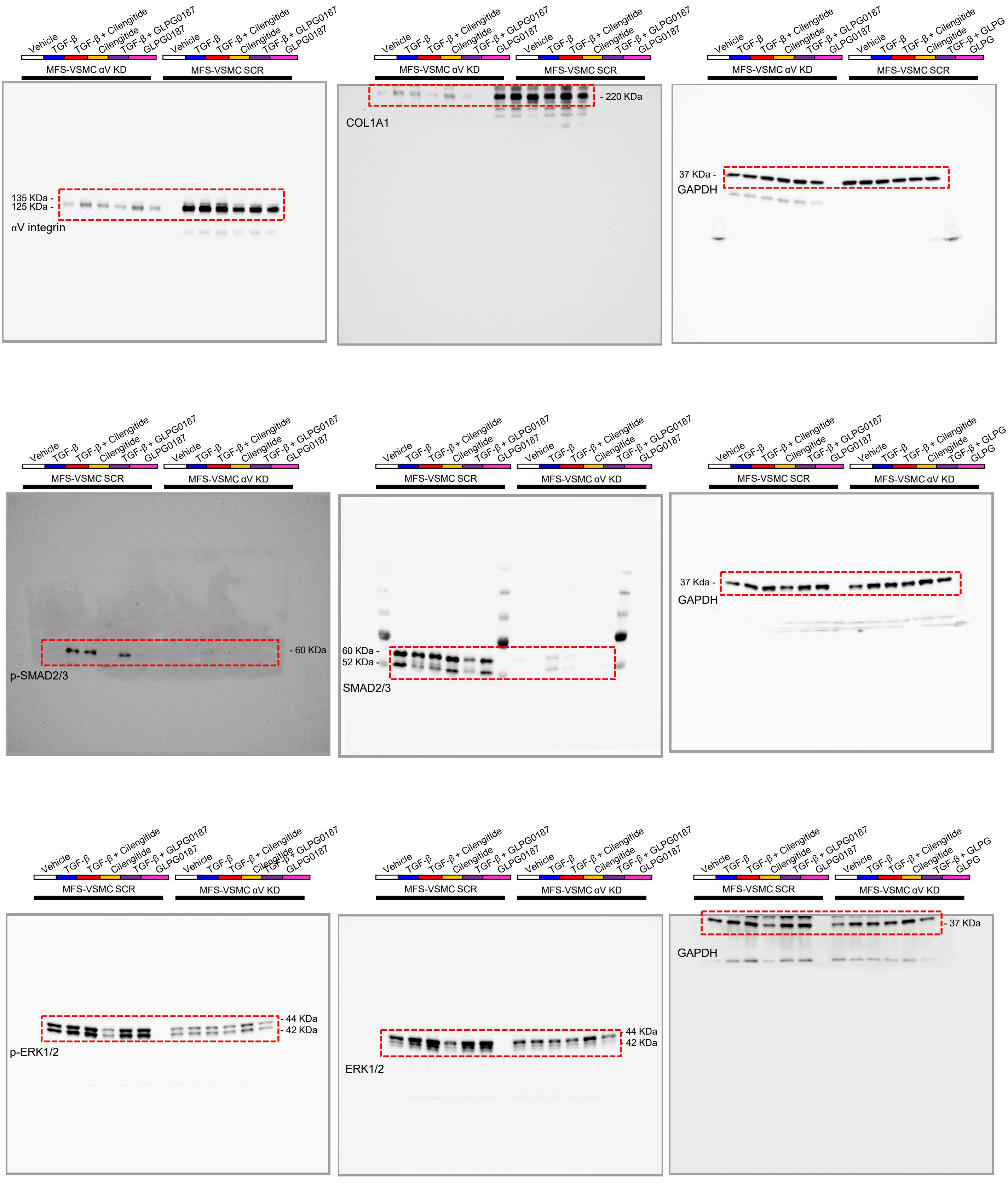

Supplement: Supplementary file 4 [file jem_20260700_sourcedatafs4.pdf]
