# Supplementary material for: Cilengitide limits syndromic and sporadic thoracic aortic aneurysm by targeting specific αV integrin
Source: J Exp Med. 2026 Nov 11;223(12):e20260700. doi: 10.1084/jem.20260700 (PMC13753961; doi:10.1084/jem.20260700)
Supplement: Supplementary file 5 [file jem_20260700_tables1.docx]

**Table S1**

**A**

| **Marfan Syndrome patients (MFS)** | | |  |  |
| --- | --- | --- | --- | --- |
| **Patient** | **Sex** | **Age at surgery** | **Comorbidities** | **Treatments** |
| M1 | F | 43 | Marfan syndrome | N/A |
| M2 | M | 24 | Marfan syndrome | N/A |
| M3 | F | 76 | Marfan syndrome | N/A |
| M4 | F | 64 | Marfan syndrome | N/A |
| M5 | M | 32 | Marfan syndrome | N/A |
| M6 | M | 34 | Marfan syndrome | N/A |
| **Sporadic non-syndromic patients (sTAA)** | | |  |  |
| **Patient** | **Sex** | **Age at surgery** | **Comorbidities** | **Treatments** |
| S1 | F | 73 | Hypertension | Beta-blockers; diuretics |
| S2 | M | 47 | CAD familiarity | N/A |
| S3 | M | 39 | N/A | N/A |
| S4 | F | 40 | Dyslipidemia | N/A |
| S5 | M | 36 | Hypertension; obesity | ACEI, calcium channel blockers; diuretics |
| S6 | F | 65 | Hypertension; obesity; CAD familiarity | Beta-blockers; diuretics; ACEI |

**B**

| **DEGs (MFS vs HC)** | | | | | |
| --- | --- | --- | --- | --- | --- |
| **Gene** | **logFC** | **logCPM** | **LR** | **PValue** | **FDR** |
| *COL1A1* | 2,70443 | 10,13416 | 7,42206 | 0,00644 | 0,04901 |
| *COL1A2* | 3,10953 | 11,94776 | 9,87448 | 0,00168 | 0,02542 |
| *COL3A1* | 2,63796 | 11,46230 | 6,10592 | 0,01347 | 0,07278 |
| *FN1* | 2,10616 | 10,41972 | 7,96449 | 0,00477 | 0,04196 |
| *CCN2* | 1,35372 | 8,20084 | 4,80758 | 0,02833 | 0,87782 |
| *PTK2* | 4,70313 | 8,79116 | 11,26450 | 0,00079 | 0,27170 |
| *RHOA* | 2,10144 | 3,01209 | 10,20020 | 0,00140 | 0,31109 |
| *ROCK1* | 1,00577 | 6,07183 | 9,05841 | 0,00261 | 0,38944 |
| *TLN1* | 1,71613 | 4,76143 | 11,49071 | 0,00070 | 0,26341 |
| *ACTA2* | 2,29146 | 8,89885 | 17,93913 | 0,00002 | 0,04491 |
| **DEGs (sTAA vs HC)** | | | | | |
| **Gene** | **logFC** | **logCPM** | **LR** | **PValue** | **FDR** |
| *COL1A1* | 2,53194 | 10,13416 | 6,83956 | 0,00892 | 0,04173 |
| *COL1A2* | 3,31949 | 11,94776 | 11,23874 | 0,00080 | 0,01039 |
| *COL3A1* | 2,57318 | 11,46230 | 6,03074 | 0,01406 | 0,05532 |
| *FN1* | 1,86726 | 10,41972 | 6,67337 | 0,00979 | 0,04413 |
| *CCN2* | 3,05430 | 12,72695 | 8,76899 | 0,00306 | 0,02178 |
| *PTK2* | 1,23393 | 3,45648 | 3,95870 | 0,04663 | 0,11872 |
| *RHOA* | 1,63125 | 9,57416 | 6,17598 | 0,01295 | 0,05280 |
| *ROCK1* | 1,12266 | 6,97864 | 5,35270 | 0,02069 | 0,07038 |
| *TLN1* | 1,88158 | 9,72179 | 6,82701 | 0,00898 | 0,04188 |
| *ACTA2* | 1,65859 | 10,25612 | 4,89412 | 0,02695 | 0,08304 |
